# Forkhead box transcription factors (FOXOs and FOXM1) in glioma: from molecular mechanisms to therapeutics

**DOI:** 10.1186/s12935-023-03090-7

**Published:** 2023-10-11

**Authors:** Peyman Tabnak, Aysa Hasanzade Bashkandi, Mohammad Ebrahimnezhad, Mahdieh Soleimani

**Affiliations:** 1 Faculty of Medicine, Tabriz University of Medical Sciences, Tabriz, Iran; 2 Imam Reza Hospital, Tabriz University of Medical Sciences, Tabriz, Iran; 3 Faculty of Medicine, Urmia University of Medical Sciences, Urmia, Iran

**Keywords:** Transcription factors, Glioma, Forkhead box protein M1, Forkhead box protein O1, Forkhead box protein O3, Noncoding RNA

## Abstract

Glioma is the most aggressive and malignant type of primary brain tumor, comprises the majority of central nervous system deaths, and is categorized into different subgroups according to its histological characteristics, including astrocytomas, oligodendrogliomas, glioblastoma multiforme (GBM), and mixed tumors. The forkhead box (FOX) transcription factors comprise a collection of proteins that play various roles in numerous complex molecular cascades and have been discovered to be differentially expressed in distinct glioma subtypes. FOXM1 and FOXOs have been recognized as crucial transcription factors in tumor cells, including glioma cells. Accumulating data indicates that FOXM1 acts as an oncogene in various types of cancers, and a significant part of studies has investigated its function in glioma. Although recent studies considered FOXO subgroups as tumor suppressors, there are pieces of evidence that they may have an oncogenic role. This review will discuss the subtle functions of FOXOs and FOXM1 in gliomas, dissecting their regulatory network with other proteins, microRNAs and their role in glioma progression, including stem cell differentiation and therapy resistance/sensitivity, alongside highlighting recent pharmacological progress for modulating their expression.

## Introduction

Glioma, a nervous system (CNS) tumor with a high recurrence rate, is responsible for 81% of adults' most primary invasive brain tumors and 30% of CNS malignancies [1]. Gliomas are classified as astrocytoma, oligodendrogliomas, ependymomas, or oligoastrocytoma based on the malignancy intensity and their histologic origination oligodendrocytic and astrocytic components of the CNS [2]. World Health Organization (WHO) categorized glioma into four grades (grades I to IV). Gliomas with WHO grades I and II are classified as low-grade gliomas (LGG). In contrast, those with grades III and IV are classified as high-grade gliomas (HGG)[3]. HGG includes several tumors consisting of glioblastoma multiforme (GBM), anaplastic oligodendroglioma (OA), and anaplastic astrocytoma (AA) [4]. The median survival time of HGGs after conventional treatments is approximately 2 to 5 years for anaplastic glioma [5] and less than 15 months for glioblastoma [6, 7]. LGGs include oligo-astrocytomas or mixed gliomas, astrocytomas, and oligodendrogliomas with an average survival rate of 7 years and eventually progress to HGGs [8]. Conventional treatments were limited to chemotherapy and radiotherapy in the past; however, despite the recent development of novel treatments such as molecular targeted therapy, stem cell therapy, immunotherapy, gene therapy, and genomic corrections, the survival rate of patients has not improved significantly in clinical settings, majorly because of low brain-blood-barrier (BBB) permeability and occurrence of the resistance to treatment [9]. Therefore, a subtle understanding of the molecular mechanisms involved in glioma progression, therapy resistance, and glioma stem cell-induced differentiation is needed for developing the efficacy of available treatments [10].

Transcription factors (TFs) play critical roles in the transcriptional processes that control gene expression; dysregulation of muted TFs is prevalent in glioma and can lead to the development of tumor-related characteristics. Various expressed TFs and their downstream targets in glioma could be utilized for therapeutic goals [11]. FOX proteins are a broad group of transcription factors that play key roles in a variety of cellular mechanisms, including cellular growth, cell differentiation, proliferation, and cell cycle control. FOX proteins are classified according to a DNA binding motif consisting of 80 to 100 amino acids, known as the FKH domain or the fork head box [12, 13]. Thus, they are categorized into 19 subtypes according to similarities in the FKH domain; despite the fact that the FOX proteins have highly similar DNA binding domains, they have diverse tissue-specific transcriptional regulation and regulatory mechanisms that allow them to perform their specialized tasks [14, 15].

FOXO is a member of the FOX family, including four subtypes (FOXO1, FOXO3, FOXO4, and FOXO6). Growth factors which are essential for stimulation of the phosphatidylinositol 3-kinase–protein kinase B (PI3K-AKT), regulate FOXO function and phosphorylation of Akt, resulting in activation of FOXO. Moreover, FOXOs are involved in various physiological and pathological mechanisms, including cell cycle arrest, apoptosis, stem cell differentiation, and oxidative stress [16, 17]. The controversial and complex regulatory functions of FOXOs in tumorigenesis have been documented. Despite their well-known tumor-suppressing properties, FOXOs can potentially induce cancer in some circumstances [18]. For instance, FOXO1s downregulation is linked to poor prognosis and decreased survival rate in myeloid leukemia (AML), soft tissue sarcoma, and breast cancer [19–21]. In contrast, the deactivation of FOXO1 in gastric cancer contributes to better outcomes, while its activation in B-cell lymphomas was shown to be associated with cancer progression [22, 23].

FOXM1 is associated with several human carcinomas, and alterations in FOXM1 signaling are correlated with carcinogenesis and oncogenesis in gliomas, prostate, lung, colorectal, breast, and hepatocellular cancers [24, 25]. In malignant glioma, abnormal FOXM1 expression has been discovered to be a prevalent molecular change. Furthermore, increased FOXM1 expression has been linked to radioresistance and poor prognosis in GBM patients [26]. In glioma, FOXM1 interacts with critical signaling pathways and molecules, including MELK, STAT proteins, Wnt/β-Catenin, growth factors, and non-coding RNAs [26–29].

Several exclusive reviews have emphasized the role of FoxM1 and FoxOs in ovarian cancer [30] and hepatocellular carcinoma [31], respectively. However, a study to do so in gliomas is missing. Therefore, we aimed to conduct this review to fill the missing gaps and shed more light on the role of these transcription factors in the pathogenesis of gliomas.

## Methods

**Fig. 1:**
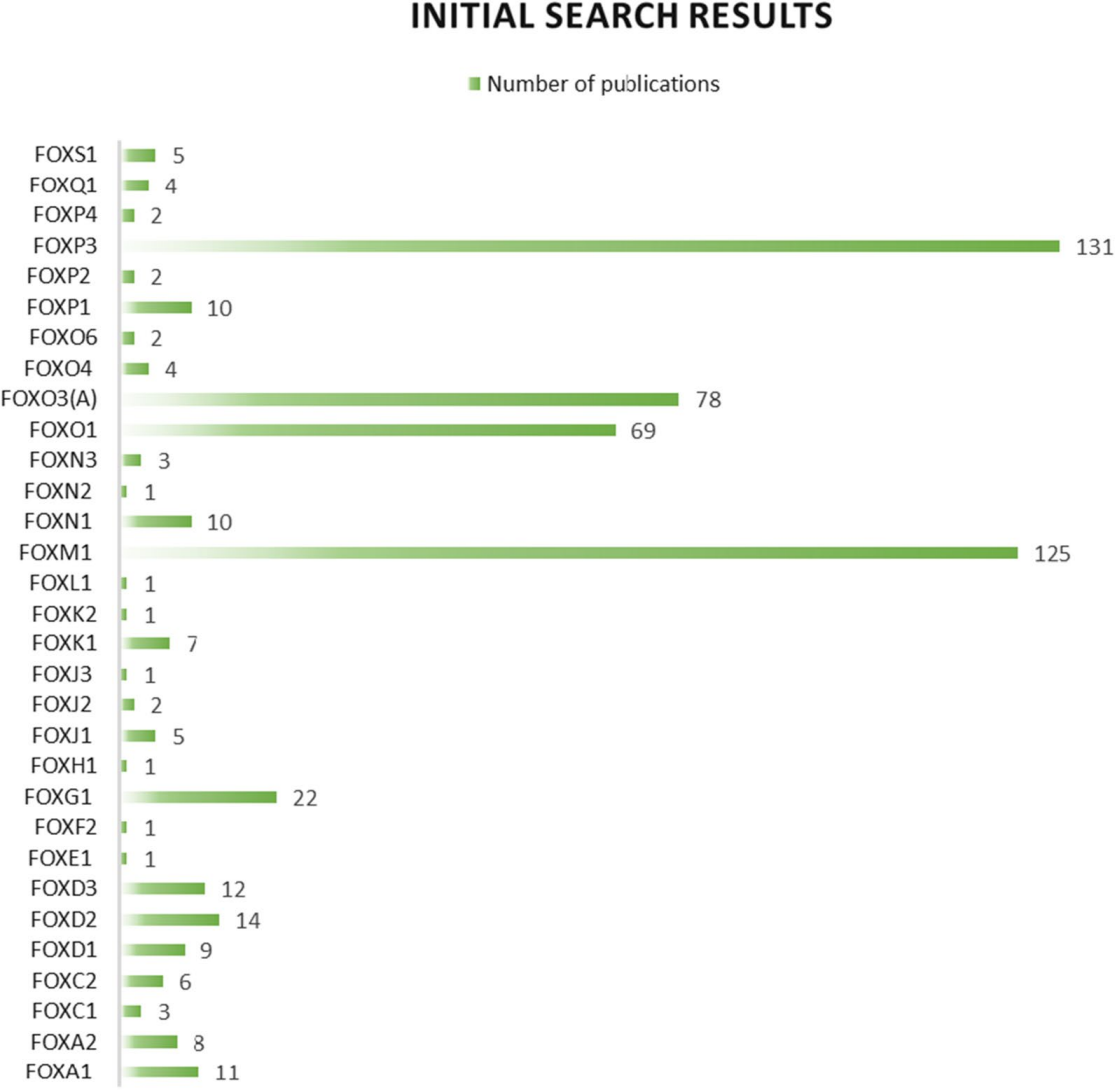
Results of preliminary search of PubMed database

**Fig. 2:**
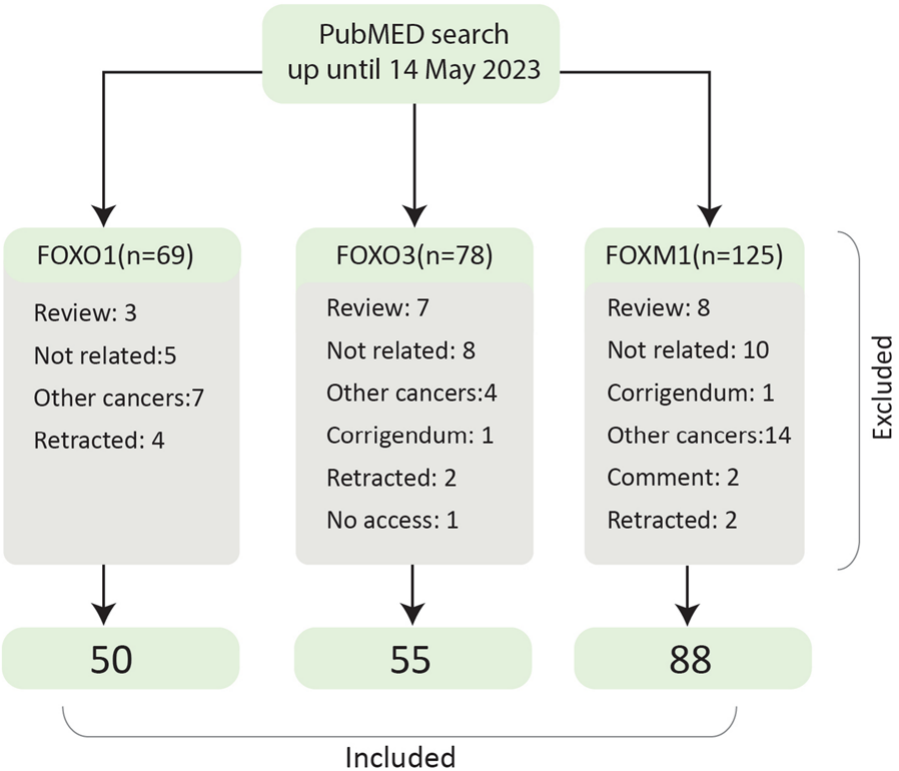
Flowchart of study selection

First, we searched PubMed on 14 May 2023 to estimate the number of published articles regarding forkhead box transcription factors in glioma using the following terms: ([Name of FOX protein]) AND (Glioma OR Glioblastoma OR Astrocytoma OR Ependymoma OR Oligodendroglioma OR Oligoastrocytoma). According to our initial assessment, the most frequently studied FOX proteins were FOXP3, followed by FOXM1, FOXO3(a), and FOXO1 (Fig. 1). However, as a subtype of regulatory T cells are also termed FOXP3+ cells, the number of studies that evaluated FOXP3 function was relatively few, leading us only to review the function of FOXM1 and FOXOs transcription factors in glioma deeply. Our inclusion criteria were original studies that evaluated these proteins' biological, prognostic, or pharmacological function in gliomas. Exclusion criteria were review articles, case reports or series, letters, editorials, consensus statements, conference abstracts, and retracted articles. The flow chart of the study selection is shown in Fig. 2.

## FoxM1

**Fig. 3:**
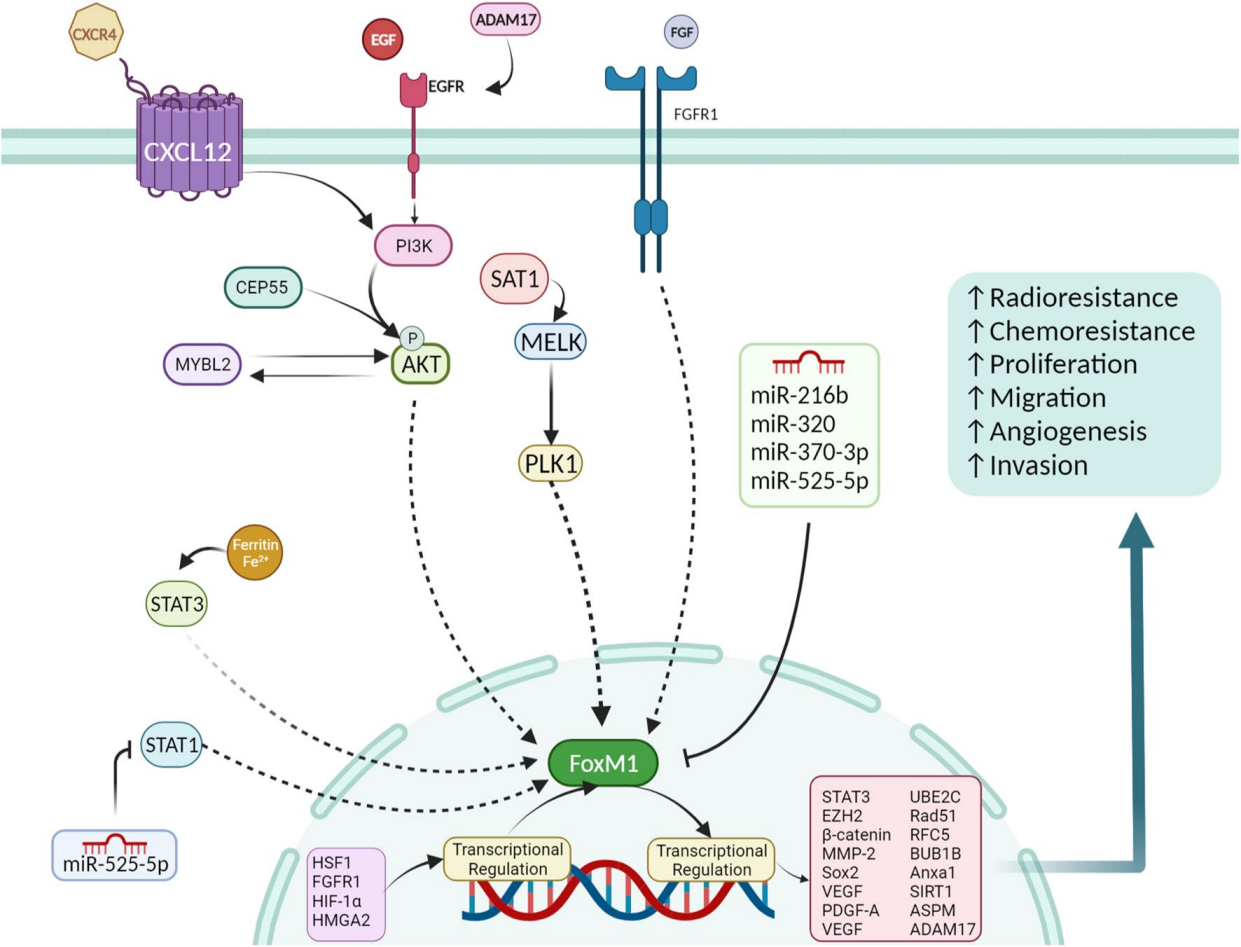
A summary of FOXM1 regulation in gliomas. Activation of growth factors and tyrosine kinases can subsequently promote FoxM1 translocation to the nucleus via inducing its translocation. In the nucleus, FOXM1 can transcriptionally regulate the expression of various targets by biding to their promoter, while several transcription factors (e.g., HSF1, FGFR1, HIF-1a, and HMGA2) can transactivate FOXM1. In addition, several miRNAs that target 3’ UTR of FOXM1 mRNA are downregulated in glioma cell lines. All of these processes lead to cell proliferation, migration, invasion, angiogenesis, as well as resistance to chemoradiotherapy

FOXM1 (forkhead box protein M1, also known as HNF-3, HFH-11, or Trident) is a transcription factor whose overexpression was implicated in the carcinogenesis of diverse tumors, especially glioma [32, 33]. This molecule is regulated at different stages of gene expression, including (a) transcriptional level (mostly cis-activated via interaction of different molecules with its binding sites and promotor), (b) post-transcriptional level (notably by non-coding RNAs: including miRNAs, lncRNAs, and circRNAs), (c) post-translational level (via mechanisms such as phosphorylation, ubiquitination, and de-ubiquitination), and (d) direct interaction of protein/RNAs with FOXM1 protein [32]. Due to this variety of targets for controlling FOXM1 expression, its inhibition seems to be a promising strategy in cancer [32]. In higher-grade gliomas, including anaplastic astrocytoma and glioblastoma, the expression of FOXM1 is significantly elevated, resulting in tumor recurrence [34–37]. In glioma tumor-initiating cells (TICs), FOXM1 is a critical factor implicated in the proliferation and self-renewal of cancer cells [38]. In this section, we will discuss the importance of FOXM1 in glioma progression, alongside mentioning its upstream and downstream regulators (Fig. 3).

### FOXM1 interplay with crucial signaling pathways and molecules in glioma

#### PI3K/AKT signaling pathway

Phosphatidylinositol 3-kinase (PI3K)/Akt, as an overactivated signaling axis, is known for contributing to the progression of malignant gliomas (GBMs) [39]. It is well established that activation of Akt can directly affect FOXM1 in solid cancers by forming a positive loop in a reciprocal manner [40]. Zhang et al. have shown that increased Akt expression can provoke FOXM1 activity. Their results demonstrated that MYB-related protein B (B-MYB/MYBL2) and FOXM1, both transcriptional factors, are co-expressed together. Their expression is strongly correlated with poor clinical outcomes and grades of gliomas. In addition, decreases in their expression can suppress glioma progression by inducing apoptosis, delay of cells in the G2 phase, and inhibiting migration, invasion, and EMT [41]. These results align with a previous study by Wang et al. which showed that binding chemokine CXCL12 to its receptor CXCR4 could significantly induce FOXM1 expression via the PI3K/AKT pathway [42]. Due to this, using a dual inhibitor of histone deacetylases (HDAC) and PI3K, such as CUDC-907, can suppress the expression and transcriptional activity of FOXM1 in high-grade gliomas, leading to radiosensitization [43].

#### MELK

Maternal embryonic leucine zipper kinase (MELK) belongs to a group of serine/threonine kinases that physiologically modulates organogenesis during the embryonic period; however, its overexpression leads to the progression of many cancers, including GBM, majorly via activating transcription factors such as FOXM1 [44]. In more detail, the activation of FOXM1 by MELK in GSCs is mediated by another kinase named PLK1. Therefore, targeting the complex composed of these proteins can be considered a desirable target in GBM [28]. The importance of the MELK/FOXM1 complex even gets bolder when EZH2, as an emerging therapeutic molecule in brain tumors [45], was confirmed to be a target of this complex in GBM spheres [46]. The MELK/FOXM1 axis has received more attention in recent years due to its significance in high-grade gliomas, and newer investigations have uncovered other upstream regulators involved in chromatin remodeling, such as SAT1 in the regulation of MELK and EZH2 [47].

#### STAT proteins

Similar to FOX proteins, STATs (signal transducers and activators of transcription) are a group of transcription factors that are mainly localized in the cytoplasm of cells; however, upon phosphorylation are translocated to the nucleus and affect target genes’ expression following the activation of cytokines (e.g., CXCR4) or growth factors (EGFR)[48, 49]. In GBM cells, FOXM1 is correlated with STAT3 levels, and inhibition of FOXM1 can prevent growth factor- and cytokine-induced STAT3 activation [50]. Schonberg et al. have found that ferritin, which stores and regulates iron ions, is preferentially expressed in GBM stem cells and associated with poor survival. They further noticed the expression of ferritin has the highest correlation with STAT3. Since FOXM1 correlates strongly with STAT3 levels, both of them can be targeted by ferritin knockdown [51, 52]. In addition, the interaction between FOXM1 and STAT3 is necessary for GBM cells’ resistance to radiation and DNA damage, which will be the point of our focus in the next parts [26]. Moreover, not only STAT3 but also STAT1 can control FOXM1 expression in different glioma cell lines (U87, A172, U251, and T98), influencing other signaling pathways implicated in inflammation, such as NF-κB [53].

#### Wnt/β-catenin signaling pathway

Wnt pathway is an evolutionary conserved pathway required for embryonic differentiation and development, and recent studies frequently addressed the consequences of its dysregulation in glioma tumorigenesis. The Canonical Wnt pathway is also referred to as the Wnt/β-Catenin pathway since it leads to the accumulation of β-Catenin in the nucleus affecting a crucial transcription factor named TCF4 responsible for Wnt target genes expression [54]. A study by Zhang et al. turned out FoxM1 acts as a downstream for canonical Wnt pathway in glioma and is required for β-catenin activation by its translocating to the nucleus, leading to self-renewal and tumorgenicity of GBM-initiating cells (GICs) [55]. More importantly, the expression of the previously mentioned protein transcription factor STAT3 is mediated by FoxM1 via enhancing β-catenin/TCF4 binding to the STAT3 gene promoter [50].

#### Growth factors

Some studies have mentioned the positive impact of FOXM1 on the expression of growth factors, including vascular endothelial growth factor (VEGF) [56] and epidermal growth factor receptor(EGFR) [57] in high-grade gliomas, all necessary for the growth and proliferation of GSCs. While FOXM1 can target growth factors expression, the receptor of growth factors such as fibroblast growth factor receptor 1 (FGFR1) has been reported to regulate the expression of FOXM1 in GBM stem cells, leading to increased expression of EMT genes, resistance to ionizing radiation, and GBM relapse after chemo-radiotherapy [58].

#### m^6^A modification pathway

One of the most common and frequent RNA modifications observed in eukaryotes is the m^6^A modification, also known as N6-methyladenosine modification. In RNA molecules, it includes attaching a methyl group to the nitrogen atom at the sixth position of the adenosine base [59]. ALKBH5 is an m6A demethylase that plays a critical role in regulating m6A modification. By removing the m6A mark from RNA molecules, ALKBH5 influences RNA stability and metabolism, consequently influencing gene expression and various biological processes in cancers [60]. In GBM stem cells (GSCs), a significantly elevated expression of ALKBH5 has been observed, which is essential for stem cell self-renewal. ALKBH5 could maintain FOXM1 mRNA stability by demethylating its nascent transcripts in GSCs, leading to tumor growth [61]. Thus, selective ALKBH5 inhibitors such as Ena15 and Ena21 are promising strategies against glioma progression as they could decrease tumor growth in different GBM cell lines [62].

#### Hedgehog signaling pathway

The hedgehog signaling system is a multidimensional molecular signaling network in animals, including humans, that plays a crucial part in embryonic development, tissue maintenance as well as cancer by controlling cell differentiation and proliferation. When hedgehog proteins attach to a receptor known as Patched, they activate another protein named Smoothened. This sets off a chain of intracellular events that activate transcription factors known as GLI proteins. GLI proteins regulate the expression of target genes in the pathway as well as other downstream signaling pathways [63]. There is evidence that GLI1 and FOXM1 are co-expressed in GBM cells. In more detail, it has been shown that FOXM1, via promoting transcription of a nuclear importer protein named IPO7, increases the nuclear localization of GLI1 proteins. The FOXM1/IPO7/GLI1 axis contributes to the proliferation, migration, and invasion of GBM cells [64]. GLI1 has a prominent role in the malignant transformation of immortalized human astrocytes [65]. These data show a positive feedback loop exists between GLI1 and FOXM1 transcription factors in different subtypes of gliomas [64, 66].

#### Other regulators and the role of non-coding RNAs

Alongside signaling pathways and molecules discussed above, various studies have indicated the relationship between FoxM1 and other molecules involved in glioma progression. As seen in Table 1, FoxM1 can regulate (upstream regulating) or be regulated (as downstream target) by a variety of molecules. Among upstream regulators of FoxM1, the prominent role of non-coding RNAs is worth mentioning. Though these classes of RNAs are not encoded into proteins, they play a significant role in epigenetic regulation of other proteins at different stages of gene expression [67]. Due to the ability of circular RNAs and long non-coding RNAs to act as a sponge for shared microRNAs at the post-transcriptional level, they are also referred to as competing endogenous RNAs (ceRNAs). In addition, the mechanism of action by which microRNAs exert their role is mostly through targeting the 3′ UTR of mRNAs [63]. Since FOXM1 acts as an oncogene in cancers and glioma is not an exception, those miRNAs whose target is FOXM1 are usually downregulated, leading to its overexpression and exacerbating glioma’s malignancy. On the other hand, the downregulation of these miRNAs is affected by oncogenic lncRNAs and circRNAs as well, which are upregulated (Table 1).

**Table 1:** Upstream regulators and targets of FOXM1 in gliomas

Name	Expression	Glioma type	Axis	Action of FOXM1	Highlights
Upstream regulators of FoxM1					
CXCL12 [42]	↑	GBM	CXCL12/AKT/FOXM1	Induces FOXM1 expression by binding to CXCR4	• ↑Cell invasion • Correlated with FOXM1 expression in human malignant glioma samples
CXCL12/CXCR4 [68]	↑	GBM	CXCL12/CXCR4/ FOXM1	-	• ↑TMZ resistance, migration, and invasion
MELK [28]	↑	GBM	MELK/FOXM1	Phosphorylation of FOXM1 is dependent on PLK1	• FOXM1 is a substrate for MELK
HSF1 [69]	↑	GBM	HSF1/FOXM1	binding sites at FOXM1 promoter	• FOXM1 is required for G2-M phase progression • Protects cells from heat shock stress • HSF1 is the critical regulator of FOXM1 expression under heat shock stress
FGFR1 [58]	↑	GBM	FGFR1/FOXM1	–	• ↑Resistance of GSCs to ionizing radiation • FGFR1/FOXM1/EMT axis is implicated in tumor relapse in patients treated with chemo-radiotherapy
α6-integrin [70]	↑	GBM	α6-integrin/ ZEB1 and YAP1/FGFR1/FOXM1	-	• ↑Proliferation, neurospheres formation, and stemness • Associated with poor prognosis
EZH2 [46]	↑	GBM	MELK/ FOXM1/EZH2	Both FOXM1 and MELK increase EZH2 promoter activity	• ↑Radioresistance of GSCs
PARP3 [71]	↑	GBM	PARP3/Rad51/FOXM1	Rad51 binds to the promoter of FOXM1/PARP3	• ↑DNA damage response • ↑Radioresistance • ↑Tumor growth in vivo • ↑Cell proliferation
NOX4 [72]	↑	GBM	NOX4/ HIF-1α /FOXM1	NOX4 induces FOXM1 by increasing mitochondrial ROS stabilization of HIF-1α	• ↑Aerobic glycolysis and proliferation • ↑Tumorigenesis in vivo
POLE2 [73]	↑	GBM	POLE2/AURKA/FOXM1	*POLE2 induces AURKA-mediated FOXM1 de-ubiquitination*	• ↑Proliferation and migration
PHGDH [74]	↑	Grade I-IV	PHGDH/FOXM1	*PHGDH stabilizes FOXM1 protein at its N-terminal*	• ↑Proliferation and invasion • Associated with poor prognosis
CEP55 [75]	↑	GBM	CEP55 /Akt /FOXM1/ MMPs	–	• Correlated with the tumor grade • Invasion migration • TMZ resistance • Neurosphere formation • Stem-like cells’ self-renewal
HMGA2 [76]	↑	GBM	HMGA2/FOXM1	HMGA2 enhances the promoter activity of FOXM1	• ↑Pericyte differentiation and invasive properties of glioma-initiating cells • ↑GIC cell-cycle progression and invasion
SAT1 [47]	↑	Grade III-IV	SAT1/MELK/FOXM1	SAT1 causes polyamine acetylation of MELK	• DNA dynamics • ↑Brain tumor stemness
TRIM56 [77]	↑	GBM	TRIM56/FOXM1/RAD51	TRIM56 enhances the stability of FOXM1 by deubiquitination	• ↓Radiosensitization • ↑ DNA repairment • Poor survival
CKAP4 [78]	↑	GBM	CKAP4/Akt/FOXM1	CKAP4 regulates FOXM1 expression through the AKT/ERK signaling pathway	• Positively correlated with tumor grade, advanced age, non-chemotherapy, IDH wildtype, non-codeletion of X1p19q, and unmethylated MGMT promoter • ↑ Proliferation, migration, and invasion of GBM cells
STEAP3 [52]	↑	GBM	TfR/STAT3/FoxM1	STEAP3 regulates FOXM1 expression through STAT3-FoxM1 axis	• ↑Cell proliferation, invasion, and sphere formation in vitro and tumor growth in vivo • Positive correlation with tumor grade (Grade III and IV)
ST3GAL1 [79]	↑	GBM	ST3GAL1 APC/C-Cdh1/FoxM1	ST3GAL1 indirectly controls FoxM1 protein degradation by the APC/C-Cdh1 complex	• ↑Cell proliferation, invasion • Correlated with patient survival, higher tumor grade, and volume
ALKBH5 [61]	↑	GBM	ALKBH5/FOXM1	ALKBH5 demethylates FOXM1 nascent transcripts, leading to enhanced FOXM1 expression	• ↑ Proliferation and tumorigenesis • Negatively correlated with patient prognosis • ALKBH5 is associated with GSC Self-Renewal
MTDH [80]	↑	GBM	MTDH/ FOXM1	MTDH stabilizes FOXM1 by inhibiting its ubiquitination MTDH enhances FOXM1 transcriptional activity	• ↑Cell cycle progression, angiogenesis, and invasion
MTDH [81]	↑	Glioma(subtype not mentioned)	MTDH/FOXM1/MYBL2	–	• ↑Cell proliferation, migration, and invasion
SGO2 [82]	↑	GBM	SGO2/ FOXM1	–	• Positively correlates with WHO grading and poor survival of high-grade gliomas • ↑Cell proliferation and migration
AVIL [83]	↑	GBM	AVIL/FOXM1	AVIL regulates FOXM1 stability through regulating F-actin dynamics	• ↑Proliferation rates and increased migration
Methionine [84]	↑	Glioma-initiating cells	Methionine/SREBF2/FOXM1	FOXM1 was down-regulated with SREBF2 by methionine depletion	• ↑ Self-renewal and pluripotency • ↑Cell proliferation • ↓Cell death • FOXM1 is associated with cholesterol metabolism
lncRNA- HULC [85]	↑	GBM and GSCs	HULC/FOXM1/AGR2/HIF-1α	HULC stabilizes the FOXM1 protein through ubiquitination	• ↑ Stemness, proliferation, and glycolysis • ↓Apoptosis
CircPIK3C2A [86]	↑	GBM	CircPIK3C2A/miR-877-5p/FOXM1	CircPIK3C2A upregulates FOXM1by sponging miR-877-5p	• ↑ Proliferation, migration, and invasion • ↑Growth of xenografted tumors in vivo
lncRNA MYCNOS [87]	↑	GBM	MYCNOS/miR-216b/FOXM1	MYCNOS upregulates FOXM1by sponging miR-216b	• ↑Proliferation
miR-216b [88]	↓	GBM	miR-216b/FOXM1	miR-216b targets 3′ UTR of FOXM1	• ↑Proliferation, migration, and invasion • ↑Tumourigenesis in vivo
miR-325-3p [89]	↓	Glioma	miR-325-3p/FOXM1	miR-325-3p targets 3′ UTR of FOXM1	• ↑Viability and proliferation growth
miR-320 [90]	↓	Glioma	miR-320/FOXM1/SIRT1	miR-320 targets 3′ UTR of FOXM1	• ↑Radioresistance caused by Sirt1 upregulation
miR-370-3p [91]	↓	GBM	miR-370-3p/FOXM1	miR-370-3p targets 3′ UTR of FOXM1	• Temozolomide sensitivity
miR-525-5p [53]	↓	Glioma	miR-525-5p/STAT1/FOXM1	miR-525-5p targets 3′ UTR of STAT1	• Development of glioma both in vitro and in vivo • Proliferation, migration, invasion, and EMT upon miR-525-5p downregulation
circBFAR [92]	↑	GBM	miR-548b/FOXM1	circBFAR sponges miR-548b	• ↑Proliferative and invasive
circCCDC66 [93]	↑	Grade III-IV	circCCDC66/miR-320ab/FoxM1	Sponging miR-320ab by circCCDC66/ Targeting 3′ UTR of FOXM1 by miR-320ab	• ↑Proliferation, migration, and invasion
Downstream targets of FOXM1					
Anxa1 [24]	↑	GBM	FOXM1/Anxa1	Enhance the Anxa1 promoter activity	• ↑Proliferation, migration, and angiogenesis
STAT3 [26]	↑	GBM	FOXM1/STAT3	–	• Radiation-induced activation of STAT3 and FOXM1 induction is mutually co-regulated • FOXM1/STAT3 interacts and co-localize following radiation therapy
CDC20 [38]	↑	GBM	FOXM1/CDC20/p21CIP1/WAF1	Binds to the promoter region of CDC20	• ↑Proliferation and survival of TICs
MYBL2 [41]	↑	Grade III-IV	Akt/ FOXM1/MYBL2	–	• Linked with poor outcome and grade • ↑Tumor progression: migration, invasion, and EMT • ↓Cell apoptosis • MYBL2 is a radiosensibility biomarker of glioma
ADAM17 [57]	↑	GBM	FOXM1/ADAM17/EGFR/Akt	Binds to the ADAM17 promoter region	• ADAM17 forms a positive loop by increasing the activity of EGFR/Akt axis • ADAM17 promotes MES transition in GBM and tumor malignancy in vivo
UBE2C [94]	↑	Grade I-IV	FOXM1/UBE2C	Binds to and activates the UBE2C promoter	• ↑Proliferation • UBE2C inhibition causes autophagy
Rad51 [95]	↑	GBM	FOXM1/Rad51	Binds to Rad51 promoter	• ↑Chemoresistance • Negative predictor of prognosis
RFC5 [96]	↑	Grade I-IV	FOXM1/RFC5	Interacts with RFC5 promoter	• FOXM1 confers TMZ resistance in glioma cells independent of MGMT activation
BUB1B [97]	↑	GBM	FOXM1/BUB1B	Binds to BUB1B promoter	• Poor prognosis in GBM • ↑Proliferation • BUB1B-mediated radioresistance is essential for GBM recurrence
Sox2 [98]	↑	GBM	FOXM1/Sox2	Binds to Sox2 promoter	• Sox2 is upregulated in radioresistant cells • FOXM1 knockdown sensitizes GBM cells to radiation • Correlates with glioma grades and predicts poor patient survival
MMP-2 [99]	↑	GBM	FOXM1/MMP-2	Binds to MMP-2 promoter	• ↑Invasion
VEGF [56]	↑	GBM	FOXM1/VEGF	Binds to VEGF promoter	• ↑Growth and angiogenesis
Sirt1 [100]	↑	Grade III-IV	FOXM1/SIRT1	Binds to the promoter region of SIRT1	-
ASPM [101]	↑	GBM	FOXM1/ASPM	Binds to the promoter region of ASPM	• ↑Proliferation, migration, and invasion • Predicts poor prognosis in gliomas
NUF2 [102]	↑	GBM	FOXM1/NUF2	Binds to NUF2 promoter	• ↑Proliferation and autophagy • ↓ Apoptosis • ↑TMZ-resistant
TRIP13 [103]	↑	Grade I-IV	FOXM1/TRIP13	–	• TRIP13 is co-expressed with FOXM1

*Exp* expression, *CXCL12* C-X-C motif chemokine ligand 12, *MELK* maternal embryonic leucine zipper kinase, *HSF1* heat shock transcription factor 1, *FGFR1* fibroblast growth factor receptor 1, *EZH2* enhancer of zeste 2 polycomb repressive complex 2 subunit, *PARP3* poly(ADP-Ribose) polymerase family member 3, *NOX4* NADPH oxidase 4, *POLE2* DNA polymerase epsilon 2 accessory subunit, *PHGDH* phosphoglycerate dehydrogenase, *CEP55* centrosomal protein 55, *HMGA2* high mobility group AT-Hook 2, *SAT1* spermidine/spermine N1-acetyltransferase 1, *MYCNOS* MYCN opposite strand, *BFAR* bifunctional apoptosis regulator, *CCDC66* coiled-coil domain containing 66, *MYBL2* MYB proto-oncogene like 2, *ADAM17* ADAM metallopeptidase domain 17, *UBE2C* ubiquitin conjugating enzyme E2 C, *RAD51* RAD51 recombinase, *RFC5* replication factor C subunit 5, *BUB1B* BUB1 mitotic checkpoint serine/threonine kinase B, *STAT3* signal transducer and activator of transcription 3, *SOX2* SRY-box transcription factor 2, *MMP2* matrix metallopeptidase 2, *VEGF* vascular endothelial growth factor, *Anxa1* annexin A1, *SIRT1* sirtuin 1, ASPM assembly factor for spindle microtubules

### FOXM1 and treatment opportunities in glioma

#### Radiotherapy

While radiotherapy combined with other treatments such as chemotherapy is considered a conventional treatment after surgical resection in high-grade gliomas, failure in treatment is frequently seen due to radioresistant exhibited by tumor cells, particularly glioma stem cells (GSCs). Various molecular pathways are involved in the radioresistance of gliomas; on top of them, there are AKT, Wnt/β-catenin, and STAT3 [104]. Surprisingly, FOXM1 is a downstream target affected by them. For this reason, FOXM1 can be considered a promising target for overcoming radiotherapy resistance in gliomas [105], as activation of the abovementioned oncogenic signaling pathways and proteins subsequently leads to the aberrant activation of this protein and radioresistance. One mechanism by which FOXM1 contributes to radiation resistance is its DNA repair capability. Cells undergoing radiation often overexpress FOXM1 to prevent further DNA damage [106]. Since FOXM1 is involved in cell cycle regulation and DNA repair, it plays a significant role in driving transcriptional response against radiation in high-grade gliomas [105]. Not only FOXM1 but also its targets also have been shown to be implicated in the radioresistance of gliomas. The previously mentioned MYBL2, as a downstream protein upregulated by AKT/FOXM1 axis, can be used as radiosensitivity biomarker for diagnosing patients with no response to radiotherapy [41]. Similarly, the expression of both STAT3 and FOXM1 was shown to be concurrent following radiation treatment in high-grade gliomas [26]. The studies emphasizing the role of FOXM1 in radioresistance in glioma have been summarized in Table 1.

#### Chemotherapy

The most commonly used chemotherapy regimens against high-grade gliomas are temozolomide (TMZ), bevacizumab, nitrosourea agents (e.g., carmustine), and platinum-based agents (e.g., cisplatin, carboplatin, and oxaliplatin). However, resistance to these drugs is commonly seen [107]. Like many other transcription factors, FOXM1 is strongly associated with the processes related to DNA repair, making glioma cells resistant to chemotherapy as well. Therefore, lowering FOXM1 has been shown to be associated with temozolomide (TMZ) sensitivity in GBM cell lines following the downregulation of DNA-repair-responsible genes such as Rad51 and RFC5 [95, 96]. Various FOXM1 inhibitors have been found in gliomas with chemosensitizing effects on in vivo and in vitro models (Table 2). For example, previous studies have found that FOXM1 can serve as a general target for proteasome inhibitors (PIs) in different cancer cell lines [108]. Bortezomib is a PI that has shown TMZ-sensitizing properties via inhibiting FOXM1 in both cellular and pre-clinical models for the treatment of high-grade gliomas [109]. However, since this agent cannot pass through the blood–brain barrier (BBB), early clinical trials generally have been accompanied by unsatisfactory outcomes, and newer generations of PIs, such as marizomib, were more successful [110]. Takei et al. have shown that GBM patients with low expression of FOXM1 had better overall survival compared to those with high levels of FOXM1 after neoadjuvant therapy with Bortezomib. Therefore, FOXM1 can be used as a biomarker for evaluating response treatment in GBM patients [111]. The treatments which target FOXM1 in glioma have been summarized in Table 2.

**Table 2:** Treatments against FOXM1 in glioma

Treatment	Cancer type	Drug type	IC50	Clinical Trial(s) for glioma	FDA approval	Target genes	Model	Highlights
Bortezomib [109]	High-grade glioma (HGG)	Proteasome inhibtor	–	NCT00611325/NCT00990652/NCT00006773/NCT00994500/NCT03643549/NCT00998010/NCT01435395/NCT00641706/NCT00544284/NCT00108069	Approved for Multiple Myeloma	FOXM1–Survivin	In vivo/in vitro	• ↓Cell viability, spheroid growth, colony formation, and stemness of glioma cells • ↑TMZ efficacy
OTSSP167 [115]	GBM	MELK inhibitor	100 to 200 nM	–	–	MELK/AKT/FOXM1 Reduce AKT phosphorylation	In vivo/in vitro	• ↓Proliferation, migration, and Invasion • ↑Cell cycle arrest in the G2/M phase • ↓Neurosphere formation in GSCs
C646 [116]	Glioma stem cells in GBM	Small molecule inhibitor of CBP	–	–	–	SATB2/CBP/FOXM1	In vivo/in vitro	• C646 inhibits SATB2/CBP transcriptional activity and inhibits tumor proliferation • SATB2 and CBP are preferentially expressed by GSCs • SATB2 recruits CBP to activate FOXM1 transcription
Fimepinostat (CUDC-907) [43]	Pediatric HGG	HDAC and PI3K inhibitor	–	NCT02909777/NCT03893487	–	decreased phosphorylation of AKT	In vivo/in vitro	• Induces cytotoxicity and synergism with radiotherapy • ↑DNA damage • ↓Expression of critical DDR genes through an NFκB- and FOXM1-mediated pathway
Siomycin A [28]	GSCs and GBM	Antibiotic	–	–	–	Abrogates FOXM1-MELK Interaction	In vivo/in vitro	• Overexpression of FOXM1 with MELK, but not FOXM1 or MELK alone, rescued the Siomycin A-induced G2/M arrest of GBM30 GSCs • Co-treatment with TMZ and Sio A is more effective than using TMZ alone
Plumbagin [117, 118]	Glioma	Natural quinonoid	2.83 μM [117] and 3.22 μM[118]	–	–	Inhibits transcriptional activity of FOXM1 ↓cyclin D1, Cdc25B, surviving ↑p21CIP1 and p27KIP1	In vitro	• ↓Viability, proliferation, migration, and invasion of glioma cells • ↑ Cell cycle arrest, apoptosis
3-O-acetyl-11-keto-β-boswellic acid(AKBA) [119]	GBM	Natural product	18.69 μM to 31.61 μM (different per cell lines)	–	–	p21/FOXM1/cyclin B1	In vivo/in vitro	• ↓Cell proliferation, DNA synthesis, migration, invasion, and colony formation • AKBA arrested the cell cycle at mitosis by regulating aurora B/TOP2A
Sophoridine [120]	Glioma	Natural product	5.3 mg/ml	–	–	Inhibits the transcriptional activity of FOXM1	In vitro	• ↓ Growth • ↑Apoptosis and arrested • Induced ROS production • Inhibited ubiquitin–proteasome
ZR30 (Human fibulin-3 protein variant) [121]	GBM	synthesized protein	–	–	–	In vivo downregulation of FOXM1 and IGFBP3 and SEMA3B EGFR/NOTCH/AKT	In vivo/in vitro	• Exerts tumor suppressive effect via downregulating membrane receptors (EGFR, NOTCH1) and their downstream AKT-signaling, MMP2, and FoxM1
Thiostrepton [96]	Glioma	Natural product	–	–	–	Inhibits FoxM1-RFC5	In vivo/in vitro	• ↓TMZ resistance and proliferation
Silibinin [122]	GBM	Natural product	–	–	–	Inhibits PI3K/Akt signaling	In vitro	• ↓Proliferation • ↑Cell apoptosis
Curcumin [66]	GBM	Natural product	15 µM and 31 uM for the U87 and T98G	NCT05768919/NCT01712542	–	Inhibits GLI1 as an upstream regulator of FOXM1	In vivo/in vitro	• ↓ Proliferation and migration • ↑G2/M Arrest and apoptosis • ↑ Survival period
Chalcone 9X [123]	GBM	Natural product	–	–	–	repressed the protein levels of FOXM1	In vivo/in vitro	• ↓Cell proliferation, apoptosis, and migration
Diferuloylsecoisolariciresinol (DFS) [124]	GBM	Natural product	–	-	-	inhibited the binding of FOXM1 and β-catenin	In vivo/in vitro	• ↓Stemness and invasiveness • ↓Viability and ATP levels • ↑ Cell cycle arrest
MeICT [125]	GBM	toxin isolated from scorpion	3.8 μM	–	–	–	In vivo/in vitro	• ↓Cell proliferation
Ena15 [62]	GBM	2OG-like ALKBH5 inhibitors	18.3 ± 1.8	–	–	Increases m6A modification of FOXM1	In vitro	• ↓Cell proliferation and cell population in the synthesis phase of the cell cycle • ↑m6A RNA level of FOXM1
Ena21 [62]			15.7 ± 1.0	–	–			

#### Immunotherapy

Immune checkpoint blockade, cytokine therapy, dendritic cell vaccines, viral therapy, and CAR-T therapy all have been tried as immunotherapeutic approaches against gliomas, and among them, immune checkpoint inhibitors and CAR-T therapy have shown promising therapeutic values in clinical trials [112]. A recent clinical trial has highlighted the efficacy of early treatment with a vaccine-based immunotherapy approach using glioma oncoantigens (GOAs) containing FOXM1 before starting chemotherapy or radiotherapy to prevent possible chemo-radio resistance [113]. It has been shown that chimeric antigen receptor (CAR) T cells with costimulatory MyD88 and CD40 (MC) endo-domains have a higher levels of FOXM1, indicating that stimulation of FOXM1 in CAR-T cells might improve the results of immunotherapy [114].

## FOXO family

The "O" subfamily of forkhead box transcription factors consists of four members, including FOXO1 (FKHR), FOXO3 (FKHRL1), FOXO4 (AFX), and FOXO6, generally considered tumor suppressors via inducing apoptosis and inhibiting proliferation. While FOXO proteins are majorly silenced following PI3K/AKT and Ras/MEK-ERK(MAPK) pathways overactivity, they can be activated by oxidative stress regulators such as JNK (c-Jun N-terminal kinase) and MST1 (Mammalian Ste20-like kinase). Moreover, varieties of tumoral processes, including invasiveness, angiogenesis, metastasis, and drug response/resistance, are dependent on their deregulation [126]. This standpoint has been revisited against previous thoughts regarding the tumor-suppressive effects of FOXOs. Multiple theories have been proposed to justify this controversy. Depending on the context, the stage in which tumor cells are plays an essential role in the consequences of FOXOs’ transcriptional output. The impact of epigenetics, concurrent signaling pathways, and spatial localization of cells in tumor spheroids were shown as different factors responsible for metastasis-promoting outputs of FOXOs expression [127]. On the one hand, the interplay between PI3K/AKT pathway and FOXOs [126], and on the other hand, the interaction with WNT/β-catenin and TGF-β pathway is supposed to be an essential factor in forming a balance between the anti-tumor and tumor-promoting activity of FOXOs [127].

### FOXO1

Post-transcriptional modifications (e.g., phosphorylation, ubiquitination, acetylation, and deacetylation) of FOXO1 were shown to play a substantial role in regulating cell proliferation, apoptosis, autophagy, and oxidative stress [128]. The controversial role of FOXO1 in tumorigenesis is also seen in gliomas. A recent study by Chen et al. has indicated the anti-tumor capacities of FOXO1 in GBMs favor prolonged cell survival and decreased migration, invasion, cell adhesion (EMT), and drug resistance to chemotherapeutic agents such as TMZ, BCNU, or cisplatin [129]. However, these findings are in contrast with their previous study that showed both nuclear and cytoplasmic FOXO1 expression is increased in astrocytomas and GBM cells, associated with poor survival [130]. Likewise, in a recent study with a small sample size by Huang et al. immune-cytoplasmic-staining scores of FOXO1a helped distinguish low-grade-gliomas from non-neoplastic lesions but did not correlate significantly with WHO grades [131]. Later, further research conducted on TCGA-LGG and GTEx brain databases showed that low-grade gliomas have a significantly upregulated FOXO1 expression. A nomogram containing this gene alongside other autophagy-related genes (e.g., GRID2, MYC, PTK6, IKBKE, BIRC5, and TP73) could predict the survival of patients with excellent accuracy (AUC: 0.81–0.90) [132]. Another study has revealed that in both GBM and lower-grade gliomas undergoing hypoxia (higher expression of HIF-1α,) the expression of FOXO1 is also elevated [133]. However, more experimental studies than bioinformatic studies are required to confirm these results. Due to this duality, for each study reviewed here, the tumor-suppressive or tumor-supportive features of FOXOs will be highlighted (Fig. 5 and Table 3).

**Table 3:** Upstream regulators and targets of FOXO1 in glioma

Name	Cancer type	Axis	Action	Role of FOXO1	Highlights
Upstream regulators of FOXO1					
Sirt1 [153]	GBM	AKT-ERK/SIRT1/FOXO1	–	TS	• ↓Proliferation and migration
TGF-β/Smad [148]	GBM	TGF-β/Smad/FOXO1/p21Cip1	Binding to FOXO1 and forming a complex	TS	• SMAD3-SMAD4-FOXO1 complex can inhibit cell proliferation by inducing p21Cip1
lncRNA-DANCR [154]	GBM	IGF2BP2/DANCR/FOXO1/PID1	DANCR elevated the ubiquitination level of FOXO1	TS	• IGF2BP2 expression is increased in GBMs, leading to lncRNA DANCR overexpression • DANCR causes etoposide resistance via inhibiting FOXO1
miR-5188 [138]	Glioma	SP1-activated PI3K/AKT/c-JUN/miR-5188/ FOXO1	Targets FOXO1 3′ UTR	TS	• miR-5188 promoted cell proliferation, the G1/S transition of the cell cycle, migration, and invasion in glioma • c-JUN binds to the promoter region of miR-5188 • c-JUN expression is increased upon PI3K/Akt signaling activation mediated by SP-1
SOX2/miR-486–5p [150]	GBM	Sox2/miR-486–5p/FOXO1	miR-486–5p targets FOXO1 3′ UTR	TS	• Sox2 induces miR-486–5p • miR-486–5p regulates GBM cell survival • Suppression of miR-486–5p in vivo inhibits the growth of GBM and sensitizes it to radiation
miR-28-5p [155]	GBM	miR-28-5p/ FOXO1/CyclinD1	Targets FOXO1 3′ UTR	TS	• ↑Growth
miR-374a [156]	Glioma	miR-374a/FOXO1/Bim and Noxa	Targets FOXO1 3′ UTR	TS	• ↓Mitochondrial apoptosis • ↑Resistance to etoposide
miR-135a [149, 157]	Glioma	miR-135a/FOXO1	Targets FOXO1 3′ UTR	Oncogene	• miR-135a inhibits cell proliferation and invasion by targeting FOXO1
lncRNA specific 5 (GAS5) [158]	Glioma stem cells (GSCs)	GAS5/miR-196a-5p/FOXO1/PID1 and MIIP	*miR-196a-5p targets FOXO1 3′ UTR *GAS5 downregulates miR-196a-5p	TS	• GAS5 and FOXO1 increase the expression of each other by forming a loop • ↓Migration, invasion, and proliferation • ↑Apoptosis
circGFRA1 [151]	Glioma	circGFRA1/ miR-99a/AKT/FOXO1	circGFRA1 downregulates miR-99a miR-99a targets FOXO1 3′ UTR	TS	• ↑Cell proliferation and migration
miR-637 [159]	High-grade glioma	miR-637/Akt1/FOXO1	miR-637 targets Akt1 3′UTR and Akt1 inhibits FOXO1	TS	• miR-637 is downregulated in gliomas • ↓Proliferation, invasion, and migration
miR-21 [160]	GBM	miR-21/FOXO1	Targets FOXO1 3′ UTR	TS	• ↑Proliferation • ↓Apoptosis
miR-183/96/182 [161]	Glioma	miR-183-96-182/FOXO1	Targets FOXO1 3′ UTR	TS	• ↑Cell survival via downregulating FOXO1 • ↓ROS production • Sensitizes cells to chemotherapy
mTORC2 [140]	GBM	mTORC2/ FOXO1/miR-34c/c-MYC	Regulates FoxO acetylation through Class IIa HDACs, independent of Akt	TS	• Combined inhibition of PI3K/Akt and mTORC2 suppresses acetylated FoxO/c-Myc signaling and promotes tumor cell death • Contributes to cell survival and Warburg’s effect
LITAF [162]	Glioma	LITAF/FOXO1/Bim, TRAIL, and FASLG	Increases FOXO1 expression [The exact mechanism was not mentioned]	TS	• LITAF expression did not affect proliferation and apoptosis, however, it promoted radiosensitivity of U251 and U373 cells via the FOXO1 pathway
KLF4 [163]	High-grade glioma	KLF4/FOXO1	KLF4 binds to FOXO1 promoter and inhibits its transcription	TS	• FOXO1 inhibits glioma cells invasion and growth • KLF4 expression inversely correlated with FOXO1 expression
RFP [164]	GBM	RFP/HDAC1/FOXO1	Inhibits FOXO1 mediated by HDAC1	TS	• RFP knockdown causes oxidative stress, and apoptosis and increases TMZ chemosensitivity by histone modification
TRIM47 [133]	Low-grade and high-grade gliomas	TRIM47/FOXO1	Degrades FOXO1 through ubiquitination	TS	• TRIM47 promotes the proliferation, migration, and invasion of glioma cells through FOXO1 ubiquitination
Quinolinate [165]	GBM	Metabolite of tryptophan	Leads to phosphorylation and degradation of Foxo1	TS	• Modulates M2 macrophage polarization • ↑Immune suppression
Downstream targets of FoxO1					
Cyclin D kinase 1 (CDK1( [146]	Glioma	FOXO1/CDK1	Phosphorylates FOXO1	TS	• ↑G2/M phase proportion • ↓Cell proliferation • ↑Apoptosis
p21Cip1 [148]	GBM	TGF-β/Smad/FOXO1/p21Cip1	FOXO1 binds to p21Cip1 promoter	TS	• FOXO and Smad proteins rapidly and specifically bind to contiguous sites on the p21Cip1 promoter and mediate p21Cip1 activation • FOXG1 suppresses p21Cip1 induction
PID1 [154]	GBM	IGF2BP2/DANCR/FOXO1/PID1	FOXO1 binds to PID1 promoter	TS	• FOXO1 decreases etoposide resistance by promoting PID1 expression
OCT4 [137]	GBM	FOXO1/OCT4	Binding to OCT4 promoter region	Oncogene	• FOXO1 increases the expression of stem cell markers such as OCT4
SOX2 [136]	GBM stem cells	FOXO1/SOX2	Binding to Sox2 promoter region	Oncogene	• FOXO1 knockdown marginally reduces the expression of stemness markers and cell death after γIR or γIR/PI-103 combination treatment
RFC2 [152]	GBM radioresistant cells	FOXO1/ RFC2	Binding to RFC2 promoter region	Oncogene	• FOXO1 positively regulates the expression of RFC2 in TMZ drug-resistant glioma cells • ↑Cell proliferation and colony formation • ↓Apoptosis

*TS* Tumor supressor, *DANCR* differentiation antagonizing non-protein coding RNA, *GAS5* growth arrest specific 5, *LITAF* lipopolysaccharide induced TNF factor, *KLF4* kruppel like factor 4, *PID1* phosphotyrosine interaction domain containing 1, *SOX2* SRY-box transcription factor 2, *RFC2* replication factor C subunit 2

### Akt and FOXO1 in glioma

Given that the PI3K/AKT/mTOR pathway is overactivated in 90 percent of GBMs and is closely related to FOXOs activity, controlling its expression can serve as an indirect approach for targeting FOXOs as well [39, 134]. However, it clearly has been established that inhibition of other signaling pathways and oncogenes should be taken into account for attaining therapeutic response. Tumor suppressor p53 has an old reputation for maintaining radiation response in different cancers [135]. Therefore, its intact activity in GBM stem cells was shown to be necessary for an adequate response to combined treatment with PI3K/mTOR inhibitors and ionizing gamma radiation followed by loss of stemness markers (e. g., SOX2, nestin, or Musashi) and FOXO1/FOXO3a decrease [136]. Another study has also shown that FOXO1 can increase the expression of a stem cell marker named OCT4, exerting an oncogenic impact in GBM cells (Fig. 5B) [137]. Moreover, the knockdown of FOXO1 was slightly able to increase response to the above-mentioned treatments, representing that for attaining higher levels of response, both FOXO1 and FOXO3a should be inhibited together. This gets more confusing when a recent study revealed that targeting FOXO1 by miR-5188 is necessary for the activation of PI3K/AKT/c-JUN signaling pathway in U87 and U251 glioma cell lines, supporting the tumor-suppressive side of FOXO1 [138]. Similarly, another study has shown that the anti-tumor features of herbal medicine named Xihuang Pill are exerted through dephosphorylation of Akt and mTOR, resulting in decreased phosphorylation of FOXO1 and its subsequent translocation to the nucleus to induce apoptosis [139]. The interplay between FOXOs and PI3K/AKT could be a possible factor in causing pro or anti-apoptotic effects; however, targeting PI3K/AKT alone is not enough to control FOXO1 expression since it can be phosphorylated independently by other upstream regulators, such as mTORC2 [140] or CDKs [141].

### FOXO1 and metabolism in glioma

FOXO1 regulates processes related to energy homeostasis and glucose metabolism under physiological conditions in organs such as the pancreas, liver, skeletal muscle, and adipose tissue [142]. Recent studies also support its role in cancer metabolism as well [18]. In GBM cells, upon either FOXO1 or PI3K/mTOR inhibition, the expression of genes involved in glycolysis, such as LDHA, is reduced. However, surprisingly when both of them are inhibited, not only LDHA but ENO1 as glycolytic genes associated with poorer survival are increased, supporting the theory that for the efficacy of PI3/mTOR inhibitors against glycolysis, the intact activity of FOXO1 is necessary [143](Fig. 5B). Masui et al. [140] have shown that mTORC2, independent of PI3K/AKT activity, suppresses FOXO1/FOXO3 activity by promoting their acetylation (Fig. 5A). Activation of mTORC2 also leads to suppression of miR-34c, a miRNA that targets c-Myc. When c-Myc is upregulated, the Warburg effect (as a hallmark of cancer) is promoted and assists cell survival. Furthermore, another study has pointed out that treatment with Progesterone (a pleiotropic steroid hormone) in GBM cells can exert anti-tumor properties and suppress glycolysis and Warburg’s effect via inhibiting GLUT1, GAPDH, and cytoplasmic activity of FOXO1 [144].

### FOXO1 and cell cycle regulation in glioma

**Fig. 4:**
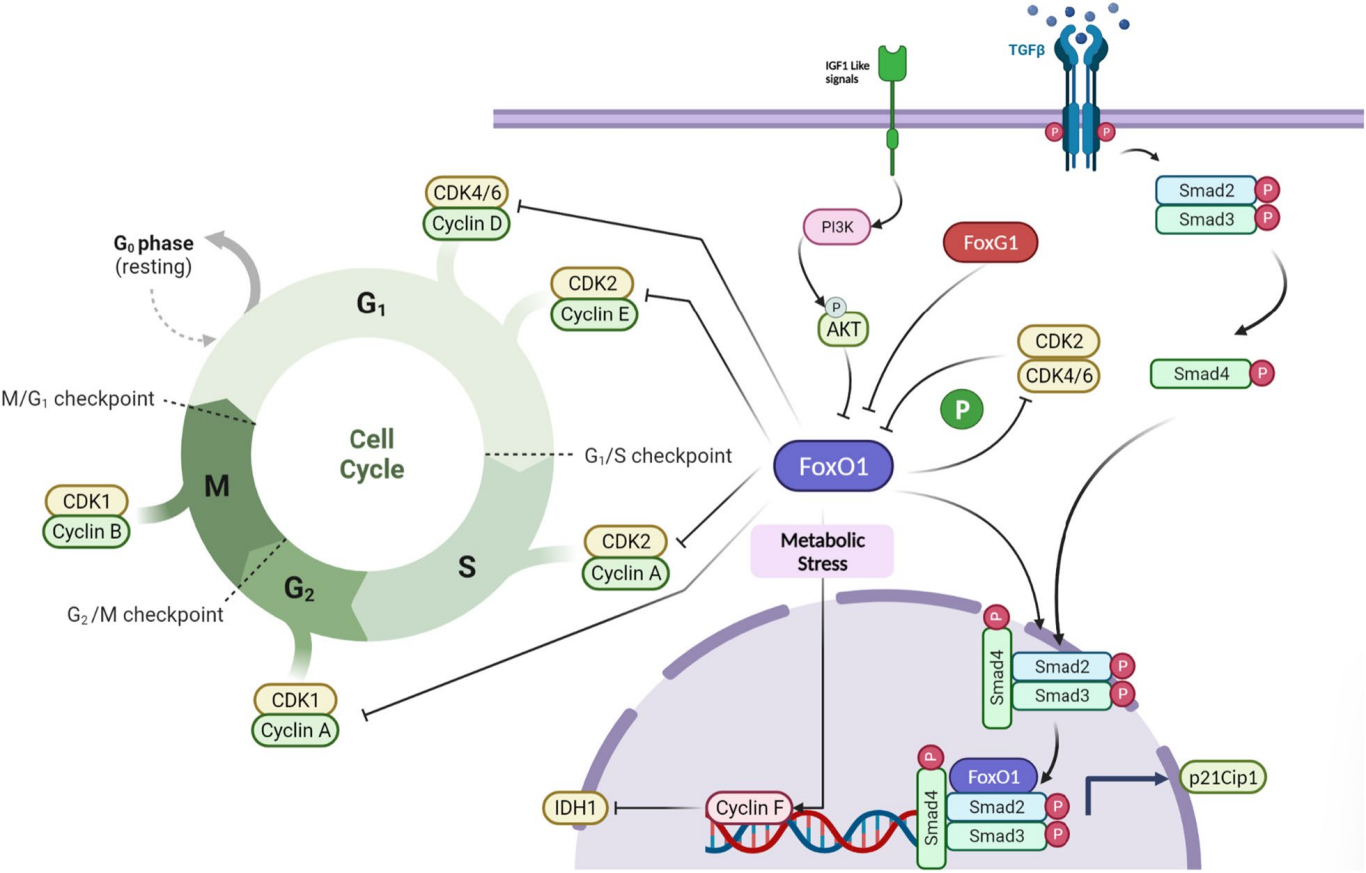
Cell cycle regulation by FOXO1 in gliomas

Regulation of the cell cycle has been proposed as an essential mechanism in which FOXO proteins exert their tumor-suppressive functions via repressing the activity of various proto-oncogenes, including cyclins (e.g., A, E, D) and cyclin-dependent kinases (CDKs including 2,4, and 6) [145]. The reciprocal interplay between cycle cell regulators and FOXOs in glioma has been reported in various studies. Restoring FOXO1 expression in gliomas can cause cell cycle arrest at the G2/M phase via phosphorylating CDK1 at s249, resulting in hindered cell proliferation and increased apoptosis [146]. Under metabolic stress conditions, cyclin F but not cyclin A, cyclin B, cyclin D, or cyclin E is expressed frequently in glioma cell lines. In more detail, the binding of FOXO1 but not FOXO3a, FOXO4, or FOXO6 to cyclin F promoter subsequently represses the expression of IDH1 as a crucial proto-oncogene in glioma [147]. In addition, it’s well established from a long time ago that cyclin-dependent kinases by phosphorylating FOXO1 on S249 cause its cytoplasmic localization and decreased activity [145]. Therefore, using CDK2 inhibitors was shown to increase the nuclear translocation of FOXO1 in U87 glioma cells more than in U251 cells [141]. Similarly, other cyclin-dependent kinase inhibitors (CDKi), such as p21Cip1, were shown to be activated by FOXO1/SMAD complex, following TGF-β signaling pathway activation in GBMs. However, PI3K/Akt signaling, as well as other forkhead transcription factors such as FOXG1, both acted as antagonists for FOXO1 by preventing TGF-β induced cytostasis(Fig. 4) [148].

FOXOs can induce cell cycle arrest at different stages by inhibiting cyclins and cyclin-dependent kinases. In addition, reciprocal phosphorylation of FOXO1 and CDKs is a crucial factor in regulating the cell cycle. Under metabolic stress, FOXO1 increases Cyclin F but not other Cyclins in gliomas, suppressing IDH1 expression, an essential tumor marker which its overexpression is implicated in glioma progression. Moreover, TFG-β/SMAD can form a complex with FOXO1 and induce the expression of CDK inhibitor p21Cip1. However, interference of PI3K/Akt signaling and other oncogenes, such as FOXG1, diminish this process.

### Other upstream regulators and downstream targets of FOXO1 in glioma

**Fig. 5:**
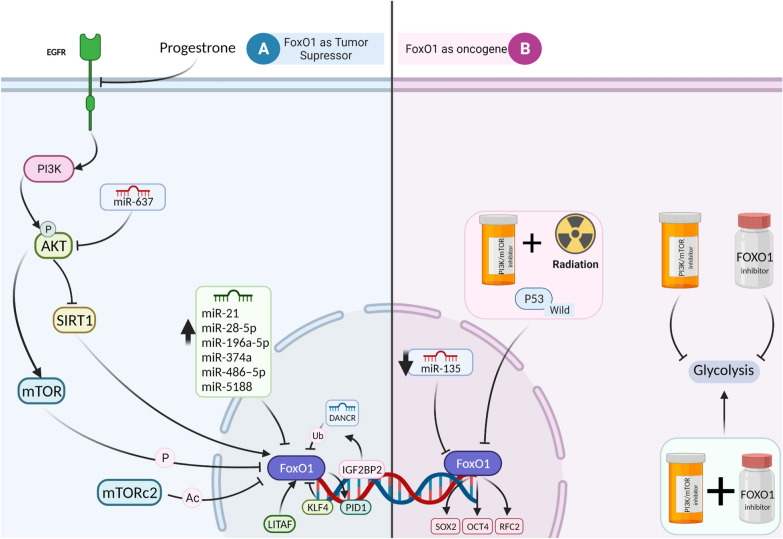
Double-edged role of FOXO1 in glioma progression. **A** Tumor suppressive effects of FOXO1. **B** Oncogenic effects of FOXO1

Similar to FOXM1, various studies have identified upstream/downstream regulators of FOXO1 in gliomas. As seen in Table 3, most of these regulators are ncRNAs, and in their results, FOXO1 was proposed as a tumor suppressor, except in a study by Shi et al. [149] which showed the opposite result. It is noteworthy to mention that some ncRNAs form a positive feedback loop that constantly represses FOXO1 expression, leading to glioma progression [138, 150, 151] (Fig. 5A). According to two other studies which proposed FOXO1 as an oncogene, two hypotheses can be raised; a) FOXO1 can act as an oncogene in radioresistant or chemoresistant glioma cell lines that have not responded to conventional treatments [152], and b) in a context-dependent manner FOXO1 can act as an oncogene by increasing stem cell markers in glioma [137] (Fig. 5B).

A)Tumor suppressive effects of FOXO1

Transduction of growth-related signals and subsequent activation of PI3K/Akt signaling cascade prevents FOXO1 translocation to the nucleus via inducing its phosphorylation. While mTOR inhibits FOXO1 translocation to the nucleus upon its phosphorylation, mTORc2, and lncRNA-DANCR have been shown to exert similar effects on FOXO1 by promoting its acetylation and ubiquitination, respectively. Moreover, treatment with agents such as Progesterone suppresses EGFR-dependent activation of the PI3K/Akt signaling pathway. There are various upregulated miRNAs, including miR-21, miR-28-5p, miR-196a-5p, miR-374a, miR-486–5p, and miR-5188 in different glioma cell lines that target 3′ UTR of FOXO1 mRNA. In the nucleus, on the one hand, FOXO1 can regulate its downstream targets (e.g., PID1). On the other hand, transcription factors such as KLF4 control FOXO1 transcriptional activity by binding to its promoter.

B) Oncogenic effects of FOXO1

Few studies have mentioned the oncogenic capabilities of FOXO1 as a therapeutic target in glioma. Following combined treatment with PI3K/mTOR inhibitor and gamma ionizing radiation, the expression of FOXO1 and stem cell marker SOX2 is decreased in GBM stem cells with wild p53 phenotype [136]. Moreover, FOXO1 can bind to the promoter of two other proto-oncogenes, including OCT4 and RFC2, and increase their transcription [137, 152]. Shi et al. have discovered that miR-135a acts as a tumor suppressor in gliomas by hindering FOXO1 expression [149]. In addition, PI3K/mTOR or FOXO1 inhibitors could prevent glycolysis in gliomas. However, when both of them are concurrently inhibited, the expression of glycolysis-related genes, including LDHA and ENO1, is elevated [143].

### FOXO1 and therapeutic opportunities in glioma

Given that many studies conducted up to now support the tumor suppressor role of FOXO1, restoring its expression may reverse glioma tumorigenesis in its early stages. Several studies have shown that restoration of FOXO1 could facilitate the efficacy of treatment with TMZ [129, 161, 164, 166], etoposide [154, 156], and radiotherapy [150, 162]. In addition, most of the pharmacological compounds which affect FOXO1 were shown to increase their expression in gliomas (Table 4). Some of these agents were shown to induce FOXO1 expression in a dose [144, 167] and/or time-dependent manner [144, 168].

**Table 4:** FOXO1 and treatment opportunities in glioma

Treatment	Cancer type	Drug type	Target genes	Role of FOXO1	Effect on FOXO1	Model	Description
Progesterone [144]	GBM	Pleiotropic neurosteroid hormone	[EGFR]/PI3K/Akt/mTOR	NA	↓↑ Time and dose-dependent	In vitro/In vivo	• ↓Tumor proliferation, angiogenesis, apoptosis
Urolithin A [153]	GBM	Natural product	ERK/AKT/Sirt1-FOXO1	TS	↑	In vitro/In vivo	• ↓Proliferation, migration and invasion • Sirt1 induces FOXO1 expression
Curcumin [169]	GBM	Natural product	CDK1/FOXO1	TS	↑	In vitro	• ↓Cell viability and proliferation • ↑G2/M cell cycle arrest and apoptosis
Caffeine [170]	GBM	Natural product	FOXO1/Bim1	TS	↑	In vitro	• ↓Cell proliferation and survival • ↑Subcellular localization of FOXO1
Xihuang pill [139]	GBM	Chinese herbal formula	ROS/Akt/mTOR/FOXO1	TS	↑	In vitro	• ↓Cell growth • Arrested cell cycle • ↑Apoptosis
PI-103 + γIR [136]	GBM-SCs with functional p53	Dual PI3K/mTOR inhibitor	FOXO1	Oncogene	↓	In vitro	• ↓Stem and progenitor cell markers as well as of FOXO1
Fenofibrate [171]	GBM	Fibric acid derivative (lipid lowering drug)	FOXO1/p27kip	TS	↑	In vitro	• ↓Cell proliferation • ↑G0/G1 arrest
Endothelial-monocyte activating polypeptide-II (EMAP-II) [167]	GBM stem cells	Tumor-derived cytokine	PI3K/Akt/FOXO1/Atg2B	TS	low dose ↑ High dose ↓	In vitro/In vivo	• ↓Tumor growth • ↑Cell cycle arrest • No effect on apoptosis • ↑Autophagy via PI3K/Akt/ FOXO1/ Atg2B axis
Zerumbone [172]	GBM	Sesquiterpenoid natural product	Akt/FOXO1	TS	↑	In vitro	• ↑Cell apoptosis through Akt inactivation and FOXO1 dephosphorylation
Trifluoperazine (TFP) [173]	SHG44 glioma cell line resistant to doxorubicin	Phenothiazine derivative (antipsychotic drug)	FOXO1	TS	↑	In vitro/In vivo	• Overcomes DOX resistance in SHG44/DOX cells via restoration of the nuclear FOXO1
NS1619 [168]	Rat brain glioma (C6) model	Calcium-activated potassium channel (Kca channel) activator	ROS/PI3K/PKB/FOXO1/ Caveolin-1	TS	↑ time dependent	In vitro/In vivo	• NS1619 increases the permeability of blood-tumor barrier due to caveolin-1 and FOXO1 expression
AU-15330 [174]	Diffuse intrinsic pontine gliomas	Degrader of the SWI/SNF ATPase subunits,	FOXO1	Oncogene	↓	In vitro	• Suppression of FOXO1 resulted in cell death in H3.3K27M cells
Denbinobin [175]	GBM	Phenanthraquinone derivative (natural product)	IKKa/Akt/FOXO1/Caspase-3	TS	↑	In vitro	• By FOXO1 dephosphorylation, leading to cell apoptosis
AS1842856 [176]	GBM	A small molecule inhibitor that inactivates unphosphorylated FOXO1	FOXO1	Oncogene	↓	In vitro	• ↓Colony formation • ↑Apoptosis

## FOXO3

Similar to FOXO1, FOXO3 (also known as FOXO3a) is generally considered a tumor suppressor in different cancers, and its sub-cellular localization was shown to be crucial for its activity. FOXO3 expression is regulated at different levels of gene expression, including post-transcriptional (mainly by miRNAs), post-translational modifications (such as phosphorylation, acetylation, methylation, ubiquitination and etc.), and protein–protein interaction [177]. As will be discussed below, the majority of studies have introduced FOXO3 as a tumor suppressor in gliomas, however; similar to FOXO1, there is a controversial role for FOXO3 in gliomas in terms of function and prognosis [178, 179]. While Qian et al. [178] have shown that in human GBM tissues, high nuclear FoxO3a expression is linked to a poor prognosis, a study with a smaller sample size by Shi et al. [179] demonstrated that in low-grade astrocytomas (grade II), the expression of FOXO3a is significantly higher than in anaplastic astrocytomas (grade III) and GBM (grade IV). However, the discrepancy in prognosis outcomes between the two studies could be attributed to factors such as sample size, patient selection, differences in FoxO3a expression levels, and the potential involvement of FoxO3a in resistance to radiotherapy and chemotherapy, which was not previously considered [178].

### Protein kinases and FOXO3 in glioma

**Fig. 6:**
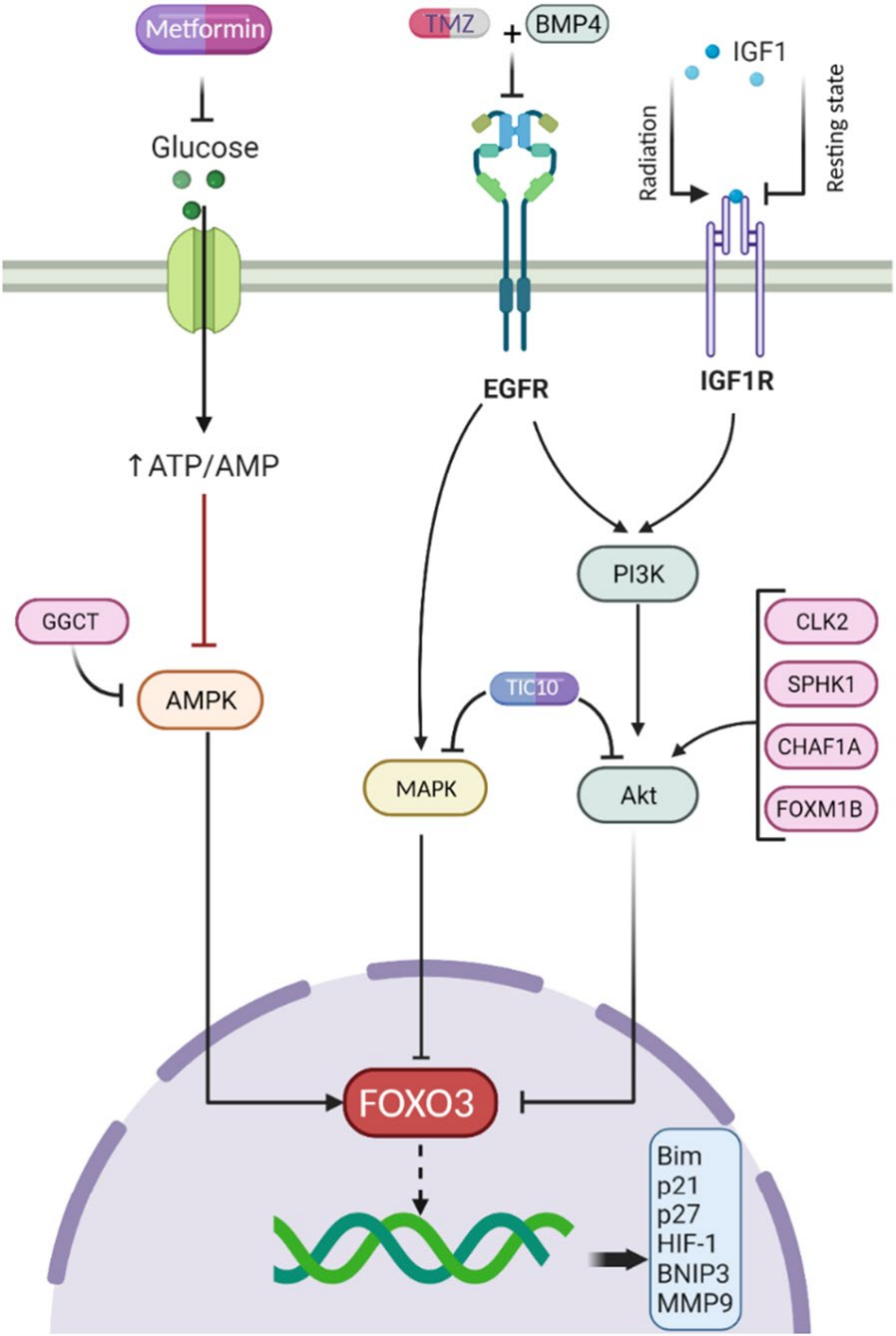
A summary of interaction between protein kinases and FOXO3 in glioma

Similar to other forkhead box transcription factors, nuclear exportation of FOXO3 is dependent on its phosphorylation by protein kinase B (PKB, Akt) as a downstream member of PI3K/Akt signaling cascade [127, 180]; however, other protein kinases such as AMPK [181, 182], EGFR [183–185], and MAPK [186] can also regulate its activity in gliomas. Accumulating evidence supports the abovementioned proteins' role in regulating FOXO3a in gliomas, and various pharmacological compounds exert their inhibitory role by affecting these axes (Table 6 and Fig. 6).

#### PKB(Akt)

Various upstream regulators of Akt such as CLK2 (oncogene) [187, 188], IGF1 (dual role) [189], SPHK1(oncogene) [190], CHAF1A (oncogene) [191], and importantly FOXM1B (oncogene) [192] was found to exert their function by affecting Akt/FOXO3 axis in gliomas. Moreover, our understanding of how PI3K/Akt inhibitors affect FOXOs is still insufficient in gliomas. While a previous study [179] has shown that using LY294002 as a PI3K/AKT inhibitor can activate FOXO3a in the nucleus, a recent study [137] has shown using NVP-BEZ235 (as another PI3K inhibitor) was not enough to induce its nuclear localization in GBM cells. Therefore, more studies are required to explore the mechanisms behind FOXO3a regulation by PI3K/Akt inhibitors.

#### EGFR

EGFR (a receptor tyrosine kinase) mutations are frequently seen in high-grade gliomas; therefore, its overexpression has prognostic importance in clinical diagnosis [193]. Although a significant correlation between EGFR and FOXO3a does not exist in GBM cell lines clinically [179], its inhibition can induce nuclear translocation of FoXO3a in GBM cells [185]. In fact, in GSCs with high expression of EGFR, FOXO3 is substantially upregulated, again supporting the hypothesis that FOXOs can induce stem cell proliferation. In contrast to cells with low EGFR expression, treatment with BMP4 (Bone morphogenic protein 4) alongside TMZ in GSCs with high EGFR triggers FOXO3a dephosphorylation and translocation to the nucleus to induce pro-apoptotic genes such as BCL2L11 [183]. These data show that the regulation of FOXO3 is complex and diverse factors are involved.

#### AMP-activated protein kinase (AMPK)

AMPK is a protein kinase sensitive to ADP and AMP changes in cells involved in energy homeostasis through switching anabolism to catabolism, and its activity has been well-studied in gliomas [194]. Given that AMPK can phosphorylate FOXO3a at Ser413, suppression of GGCT can be considered a promising strategy to promote the AMPK/FOXO3a/p21 axis and inhibit the proliferation of A172 GBM cells [182]. Moreover, activating the AMPK/FOXO3a axis by metformin was a desirable therapeutic strategy to prevent self-renewal and tumor formation of stem-like glioma-initiating cells [181].

#### MAPK

Activation of MAPK cascade, known as RAS/RAF/MEK/ERK signaling axis, has significant participation in gliomagenesis and tumor progression via inducing cell proliferation, metastasis, angiogenesis, and inhibition of apoptosis [195]. Sato, Sunayama, and colleagues have shown that concurrent inhibition of this pathway and PI3K/Akt/mTOR induces differentiation of undifferentiated glioma stem-like cells via activating FoxO3a transcriptional activity [196]. Their further investigation also highlighted the ROS-dependent mechanism of p38 MAPK/FOXO3 activation in GICs [186]. In addition, tumor necrosis factor related apoptosis inducing ligand (TRAIL) is a naturally occurring protein with tumor-suppressing features in various cancer cell lines. However, it suffers from efficient delivery to the brain tissue due to its chemical structure limitation for passing through the blood–brain barrier. Allen et al. could induce TRAIN expression in mice models with GBM significantly by deploying a novel TRAIL-inducing compound 10 (TIC10), and this mechanism was attributed to stimulant inhibition of Akt and ERK signaling pathways and subsequent transcriptional activity promotion of FOXO3a by TIC10 [197].

### Cellular stress and FOXO3 in glioma

FOXO3a subcellular localization and its post-translational modification are highly dependent on a wide range of stress-related conditions, including starvation, oxidative stress, hypoxia, heat shock, and DNA damage. Energetic stress often affects FOXO3a phosphorylation through activators such as AMPK and Sirt-1 as well as suppressors like Akt and CREB binding protein and p300 (CBP/p300) signals. Furthermore, under oxidative or genotoxic stress, the MEK/ERK pathway (as a downstream member of MAPK signaling) regulates mitochondrial accumulation of degraded FOXO3a and cellular respiration [198].

#### ROS, hypoxia, and nutrition starvation

**Fig. 7:**
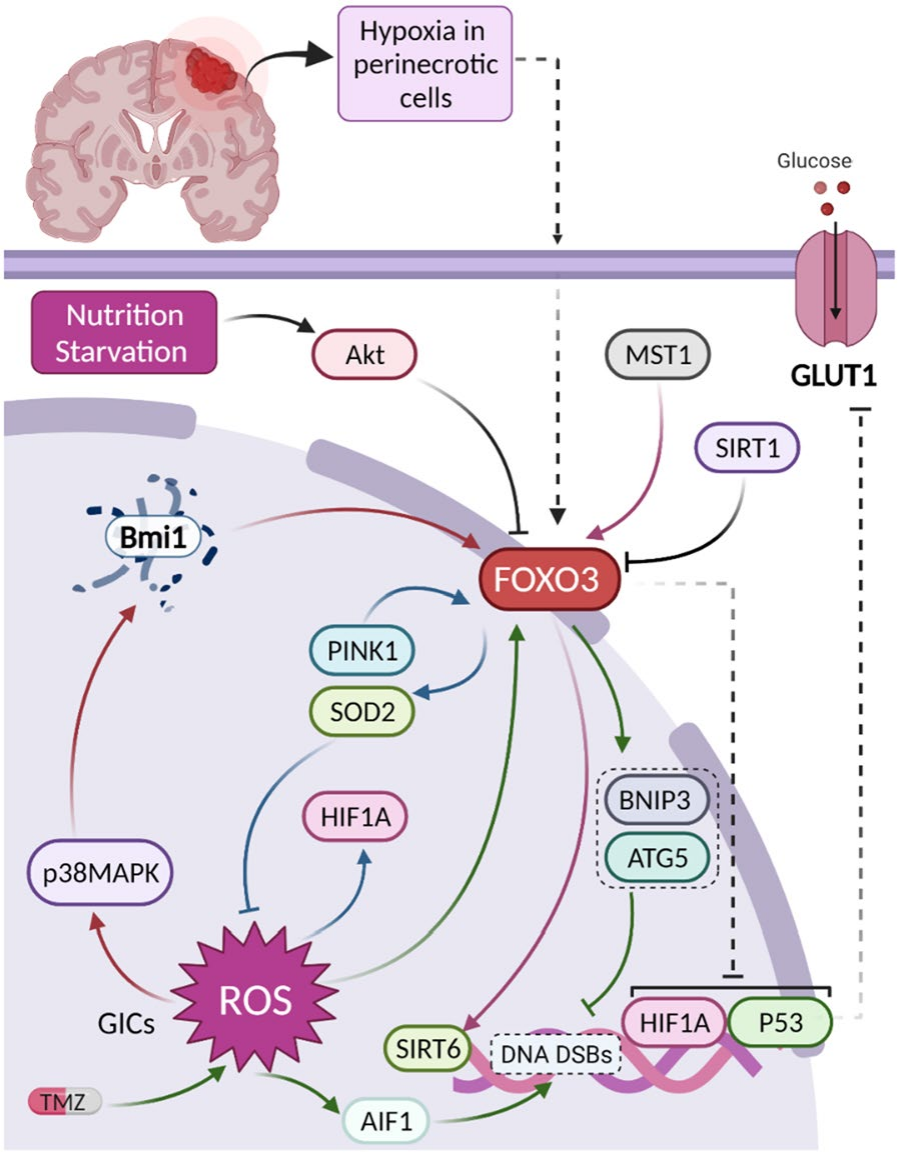
FOXO3 as a core component in regulating cellular stress: Various cellular stress conditions, including nutrition starvation, hypoxia, oxidative stress, and glucose metabolism, have a reciprocal relationship with FOXO3 in glioma. Nutrition starvation via activating Akt and deactivating FOXO3 causes radioresistance of glioma tumors. The relationship between ROS and FOXO3 is very complicated, and several molecules are involved. In GICs, the production of ROS induces p38-AMPK that, via degradation of Bmi1, activates FOXO3, resulting in differentiation and loss of self-renewal (red arrows). However, the role of ROS was shown to be double-edged in tumor progression in a way that their inhibition following PINK1-induced FOXO3 expression represses cell growth and prevents HIF-1α stabilization (blue lines). On the other hand, the study by He et al. showed that although TMZ, through producing ROS and inducing AIF1 expression, causes cell death followed by DNA double-strand breaks, in this condition, FOXO3 is activated and via upregulating BNIP3 and ATG5 prevents DNA from damage, therefore reverses this process (green lines). In addition, cells undergoing hypoxia in perinecrotic areas express FOXO3 more frequently, inhibiting the transcriptional activity of HIF-1α and p53. Since p53 can repress GLUT1 expression (a glucose transporter), inhibition of p53 by FOXO3 increases glucose consumption by tumor cells (dashed lines)

As mentioned earlier, hydrogen peroxide as a ROS can activate p38-MAPK and induce FOXO3a expression in GICs very efficiently, leading to cell differentiation and inhibited stem cell self-renewal capacity [186]. PTEN-induced kinase 1 (PINK1) negatively regulates GBM growth through activating FOXO3a and alleviating ROS and metabolic reprogramming while its loss promotes aerobic glycolysis (Warburg effect) via stabilizing HIF1α, a master modulator of hypoxia [199]. This evidence shows that FOXO3a is involved in the regulation of hypoxia. However, Hashimoto et al. have demonstrated that severe hypoxia, instead of affecting FoxO3a, increases the expression of Sp1. In addition, under hypoxic conditions, the knockdown of FOXO3a does not influence the activity of AMPK in both T98G and A172 GBM cells but suppresses Sp1 only in T98G cells [200]. Moreover, the authors have previously shown that nutrition starvation activates Akt in T98G GBM cells and slightly decreases FOXO3a expression, leading to radio-resistance. They also showed that DNA-PKcs act as an upstream regulator for FOXO3a and Akt under starvation conditions. Targeting DNA-PKcs by NU7026 can suppress their activation and slightly increase FOXO3a expression [201]. Intriguingly, Brucker et al. [202] have shown that FOXO3a expression is positively correlated with glioma WHO grade in peri-necrotic tumor lesions (where there is higher cellular stress) and under hypoxic conditions independent of HIF-1α, its upregulation causes cell death in GBM LNT-229 cells in a caspase-independent manner. More interestingly, when the FOXO3a gene was silenced, the intracellular level of ROS was significantly increased and facilitated cell death, followed by oxidative stress. Although silencing this gene saves glucose, but does not have an impact on cell proliferation. Moreover, abolishing FOXO3a lowers oxygen consumption to compensate for decreased glucose uptake of LNT-229 cells and reinforces the transcriptional activity of HIF-1α under hypoxia. More importantly, overactivation of Tp53 activity in cells with inhibited FOXO3 can improve cell survival in cellular stress conditions. These data showed that although FOXO3 is increased and results in cell death in perinecrotic tumors undergoing hypoxia, silencing its expression can also accelerate cell death via promoting excessive ROS production (Fig. 7). Taken together, several conclusions can be obtained from these studies regarding the different aspects of FOXO3 in gliomas: (a) The expression and function of FOXO3 differ in glioma depending on the spatial localization of tumor cells and tumors grade. (b) Cells tolerating hypoxia tend to promote FOXO3 activity. (c) Although FOXO3 can regulate hypoxia, there are more crucial modulators of hypoxia, such as HIF-1α, AMPK, Sp1, and Tp53. d) Role of FOXO3 in modulating oxidative stress is complicated and is highly dependent on the amount of available ROS produced by other regulators in tumor cells. (e) Under hypoxia, FOXO3 can regulate HIF1a, but the opposite is not true [202].

#### DNA damage

Repairing DNA at the G2-M checkpoint was shown to be stimulated by FOXO3a in mammalian cells [203]. In line with this, He et al. [204] have reported that, on the one hand, TMZ causes the production of mitochondrial superoxide (ROS) that subsequently, via increasing apoptosis-inducing factor (AIF), induces cell death. On the other hand, the excessively produced ROS elevates FOXO3a expression and gradually promotes BNIP3 and ATG5, two autophagy-related genes, and resulted in resistance to TMZ-induced DNA double-strand breaks (DSBs) caused by mitophagy.

#### Sirtuins

Sirtuins are deacetylase enzymes dependent on NAD^+^ for regulating cellular metabolism in response to stress. Dysfunction of sirtuins leads to various diseases, including cancer and neurodegeneration. Like FOXO transcription factors, these proteins have a dual oncogenic and tumor-suppressive function via regulating DNA repair, transcriptional modulation, and metabolism context-dependent depending on tissue type and cancer type [205, 206]. SIRT6 is a nuclear-residing protein that, due to its deacetylase activity, affects a variety of targets and substrates, including FOXO3, PARP1, MYC, and HIF-1α, involved in metabolism and chromatin/DNA repair [205]. MST1 is a downregulated protein kinase in GBM cells that inhibits cell viability, colony formation, and aerobic glycolysis but exerts apoptotic effects via directly increasing FOXO3a expression and its proposed downstream target SIRT6 [207, 208]. In contrast, SIRT1 was shown to inhibit acetylation of FOXO3a; however, treatment with betulinic acid (BA) as a natural pentacyclic triterpenoid could induce FOXO3a via repressing SIRT1, leading to mitochondrial dysfunction and cell death [209].

#### β-catenin and FOXO3 in glioma

Β-catenin is a protein with multiple functions that plays an important role in Wnt signal transduction pathway via regulating gene transcription and cell adhesion [210]. Upon β-catenin proteins translocation to the nucleus they form a complex with binding to transcription factors named lymphoid enhancer factor/T cell factor (LEF/TCF), activating the target genes of Wnt signaling pathway [210]. FOXO proteins (especially FOXO3a) were shown to compete with TCF for binding to β-catenin and suppress TCF transcriptional activity particularly under oxidative stress [211]. In line with the study conducted by Xu and colleagues [212], Sun et al. demonstrated that in U87 and U251 GBM cells resistant to TMZ, overexpression of FOXO3a positively regulates the amount of nuclear β-catenin via governing MMP9 expression [213]. In contrast, Lu et al. have shown that miR-370 as a downregulated tumor suppressor miRNA by targeting 3′ UTR of β-catenin mRNA, suppresses its expression in astrocytoma and GBM cells and subsequently promoting FOXO3a nuclear accumulation, suppressing cancer cell proliferation [214]. These data again support the oncogenic activity of FOXO proteins in therapy resistant cell lines.

#### Cell cycle regulation and FOXO3 in glioma.

Studies have reported that FOXO3 can control cell cycle via increasing transcriptional activity of two important pro-apoptotic genes including BIM [183, 190, 191, 209, 215–217]and p27 [184, 187, 188, 218–220] in gliomas, emphasizing the tumor suppressive impact of Akt/FOXO3a/BIM axis [183, 190, 191]. In addition, other transcription factors including SOX2 and FOXG1 can repress FOXO3a expression level in GBM stem cells leading to cell cycle re-entry and dedifferentiation [221].

#### FOXO3’ function in glioma stem cells

An important function of FOXO3 is its contribution to stem cell differentiation in both neural stem cells and glioma stem cells [222]. It has been proposed that nuclear accumulation of FOXO3a in GBM cancer stem-like cells could induce their differentiation. Due to the prominent role of PI3K/Akt/mTOR and MEK/ERK signaling pathways in the phosphorylation of FOXO3a, inhibiting these two signaling pathways can be an promising method for differentiation therapy against high-grade gliomas, especially GBM [137, 196, 221]. However, once GBM cells undergo chemotherapy and radiotherapy and maintain resistance to these treatments, FOXO3a overexpression exerts oncogenic function by increasing the expression of stem cell markers such as SOX2 [136]. A study suggests that following *repeated radiation*, continuous IGF1 stimulation ultimately induces FoxO3a activation, leading to slower proliferation and enhanced self-renewal. In contrast, after *acute radiation* in GBM stem cells, IGF1R/AKT/FOXO3a axis induce radioresistance [189]. It should be noted that the activity of FOXO proteins is highly dependent on the other upstream regulators. For example, BMP4 treatment only is effective in sensitizing those glioma stem cells with high EGFR expression to TMZ treatment, leading to the accumulation of FOXO3a in the nucleus [183]. As mentioned above, following radiotherapy and chemotherapy, FOXO3a induces the expression of stem cell markers. Therefore, the knockdown of FOXO3a in glioblastoma multiforme stem cells with intact p53 activity can significantly enhance the response to treatment with radiation therapy combined with PI3K/mTOR inhibition [136].

#### Regulation of FOXO3 by non-coding RNAs

Several studies have reported that 3′ UTR of FOXO3a is targeted by oncogenic microRNAs, including miR-10b [223], miR-27a [224], miR-93 [225], miR-155 [226], and miR-184 [218] that their expression is upregulated in glioma cell lines (Table 5). In addition, FOXO3a can also mediate the expression of non-coding RNAs. Temozolomide-associated lncRNA (lnc-TALC) is an overexpressed lncRNA in TMZ resistant cell lines that upregulates c-MET through competitively binding to its regulator miR-20b-3p. c-MET can promote cytoplasmic degradation of FOXO3a via activating Akt signaling pathway. In TMZ sensitive cell lines, there is much more nuclear levels of FOXO3a compared to resistant cells, which through binding to promoter of lnc-TALC inhibits its expression and results in MGMT silencing [227].

**Table 5:** Upstream regulators and targets of FOXO3 in glioma

Name	Cancer type	Axis	Role of FOXO3 in the study	Action	Highlights
CHAF1A [191]	GBM	CHAF1A/AKT/FOXO3a/Bim	TS	CHAF1A phosphorylates Akt and FOXO3a and decrease its nuclear localization	• ↓Survival and apoptosis • ↑Proliferation
IGF1 [189]	GSCs	IGF1/AKT/FOXO3a	Oncogene in radioresistant tumor spheres/TS in non-radiated cells	IGF1 induces FOXO3a expression in the resting state and nuclear localization of FOXO3a in radioresistant cell lines	• In the resting state, IGF1 downregulates Akt and FOXO3a activation, which results in slower proliferation and enhanced self-renewal • After acute radiation, IGF1 promote a rapid shift from a latent state toward activation of Akt survival signaling, resulting in GSCs radioprotection
GGCT [182]	GBM cells	AMPK/FOXO3a/p21	TS	GGCT inhibits AMPK, and AMPK phosphorylates FOXO3a at Ser413	• ↑Growth
Cathepsin B and uPAR [184]	glioma xenograft cell lines	CathB-uPAR/EGFR/Akt-ERK/FOXO3a/p27	TS	CathB-uPAR decreases the activity of FOXO3a	• ↑Cell proliferation
CLK2 [188]	GSCs	CLK2/AKT/FOXO3a	TS	CLK2 phosphorylates Akt and FOXO3a and decrease its nuclear localization	• ↑Proliferation and viability via inhibiting cell cycle arrest • ↓Survival and outcome
SPHK1 [190]	Glioma	SPHK1/Akt/FOXO3a/Bim	TS	SPHK1 phosphorylates Akt and FOXO3a and decrease its nuclear localization	• ↓Apoptosis
FOXM1B [192]	GBM	FOXM1B/NEDD4-1/PTEN/Akt/FOXO3a	TS	PTEN reduction and Akt activation	• ↑Malignant transformation • ↑Growth
FOXG1/SOX2 [221]	GBM stem cells	FOXG1/SOX2/FOXO3	TS	Transcriptional repression	• FOXG1 reduces BMP-induced astrocyte differentiation • SOX2 is necessary for constant proliferation but FOXG1 is not
PINK1 [199]	GBM	PINK1/FOXO3a/ROS/HIF-1	TS	PINK1 inhibits FOXO3a phosphorylation	• ↓Viability and growth
DNA-PKcs [201]	GBM	DNA-PKcs/FOXO3a	NA	NA	• DNA-PKcs is activated under nutrient starvation and activates Akt, MST1, and FOXO3a
MST1 [208]	Glioma	MST1/FOXO3a/SIRT6	TS	MST1 translocated FOXO3a to the nucleus	• ↓Cell viability and colony formation and induces cell apoptosis
PTEN [228]	GBM	PTEN/Akt/ FOXO3a/LIFRβ/STAT3	TS	PTEN loss and consequent Akt activation inhibit FOXO3-dependent LIFRβ gene expression in astrocytes	• PTEN loss correlates tightly with low levels of LIFRβ expression and inactivation of STAT3
Circ-DONSON [229]	Glioma	Circ-DONSON/FOXO3a	TS	Downregulates FOXO3a	• Circ-DONSON was correlated with lymph node, distant metastasis, and poor prognosis of glioma patients • ↑Cell proliferation and migration
miR-27a [224]	GBM	miR-27a/FOXO3a	TS	Targets 3′UTR of FOXO3a	• ↑Proliferation and growth
miR-155 [230]	Murine glioma and CD8+ cells	miR-155/FOXO3a/Akt and Stat5	NA	Targets 3′UTR of FOXO3a	• miR-155 loss induced glioma progression, reduced the CD-8^+^ T Cells • FoxO3a negatively regulates Akt and STAT5 expression
miR-155 [226]	Glioma	miR-155/FOXO3a	TS	Targets 3′UTR of FOXO3a	• ↑Proliferation by inhibiting apoptosis • ↑Migration and invasion
miR‑370 [214]	Astrocytoma and GBM	miR‑370/β-catenin and FOXO3a	TS	Probably targets 3′UTR of FOXO3a	• miR-370 mimics leads to accumulation of FOXO3a in the nuclei astrocytoma cells
miR-93 [225]	Glioma	miR-93/ PTEN, PHLPP2 and FOXO3	TS	Targets 3′UTR of FOXO3a	• ↑Proliferation and cell cycle progression through suppressing PTEN, PHLPP2 or FOXO3
miR-184 [218]	Glioma	miR-184/FOXO3a	TS	Targets 3′UTR of FOXO3a	• ↑Proliferation
miR-10b [223]	GBM	miR-10b/FOXO3a	TS	NA	• ↑Angiogenesis • Poorer patient survival
miR-27a-3p [231]	GBM	miR-27a-3p/FTO/FOXO3a	TS	miR-27a-3p targets 3′UTR of FTO FTO interacts with FOXO3 to increase its expression	• ↑Proliferation, invasion, migration, and tumor growth
Downstream targets					
BNIP3 [204]	Glioma	TMZ/ROS/FOXO3a/BNIP3	Oncogene	ROS produced by TMZ induces expression of FOXO3a, which causes BNIP3 overexpression	• ↑TMZ-induced mitophagy • Protects cells against temozolomide-induced DNA double strand breaks
SIRT6 [207]	GBM	FOXO3a/SIRT6	TS	FOXO3a positively correlates with SIRT6 expression	• FKHRL1/FOXO3a low expression predicts poor prognosis of patients with glioma • ↓Warburg effect and cell proliferation • FOXO3a negatively correlated with glycolytic genes including glut4, glut1, and LDHA
MMP9 and β-catenin [212, 232]	Glioma cells resistant to TMZ	FOXO3a/MMP9/β-catenin	Oncogene	FOXO3a induces β-catenin via increasing MMP-9 expression	• Poor prognosis
Lnc-TALC [227]	GBM	Lnc-TALC/miR-20b-3p/c-Met/MGMT	TS	FOXO3 binds to the promoter region of lnc-TALC in TMZ sensitive cells In TMZ resistant cells degradation of FOXO3a followed by c-Met/Akt axis in cytoplasm is observed	• Less aggregated FOXO3 was in the nucleus of TMZ-resistant GBM cells than in parental cells • Knockdown of AKT in TMZ-resistant cells decreased lnc-TALC but increased the level and nuclear translocation of FOXO3

#### FOXO3a and therapeutic opportunities in glioma

Though the mechanism of many drugs on FOXO3 has been mentioned above, current conducted pharmacologic research with relying on its tumor suppressive properties has shown remarkable results. These treatments mainly include PI3K/mTOR inhibitors, metabolism related drugs (e.g., metformin and Fenofibrate), and natural derived compounds. However, as seen in Table 6, a limitation of these studies is that they are limited to in vitro studies rather than pre-clinical clinical or levels. Moreover, designing strategies against oncogenic activity of FOXO3 can be a step forward.

**Table 6:** FOXO3 and treatment opportunities in glioma

Treatment	Cancer type and cell line	Drug type	Target genes	Role of FOXO3	Effect on FOXO3	Model	Description
Metformin [181, 233]	Stem-Like Glioma-Initiating Cells	Biguanide for diabetes type 2	AMPK/FOXO3	TS	By increasing AMPK, activates FOXO3	In vivo/In vitro	• Differentiation of stem-like glioma-initiating cells • ↓Tumor-Initiating Potential
BMP4 + TMZ [183]	GBM GSC	Chemotherapy agent and natural protein	EGFR/AKT/FOXO3a/BIM	TS	Inhibition of Akt causes FOXO3 translocation in high EGFR cells	In vitro	• ↑Differentiation and • apoptosis in high expressing EGFR cells
GSK2126458 + LY2874455 [187]	GSC	PI3K/mTOR and FGFR inhibitors	14–3-3τ/CLK2/AKT/FOXO3a/p27	TS	CLK2 regulates AKT phosphorylation through PP2A activity	In vivo/In vitro	• ↑ Sub-G1 phase, suggesting apoptosis induction • Depletion of CLK2 enhanced the effect of FGFR inhibitors in GSCs
LY294002 [179]	GBM	PI3K pharmacological inhibitor	AKT/FOXO3a/p27	TS	FOXO3a nuclear translocation	In vitro	• G1 arrest through retention of activated FOXO3a
NVP-BEZ235 [137]	GBM	PI3K pharmacological inhibitor	AKT/FOXO3/ OCT4 and SOX2	TS	FOXO3a nuclear translocation and binding to OCT4 promoter	In vitro	• Induced OCT4 in glioblastoma cells with intact FOXO3 activity, not mutant cells
Erlotinib and trifluoperazine [185]	GBM	EGFR inhibitor and FOXO3 activator	EGFR/Akt/FOXO3a/p27	TS	Inhibits EGFR and FoxO3a phosphorylation	In vitro	• Erlotinib induces FOXO3a dephosphorylation and nuclear accumulation • ↓Growth • Their combination synergistically reduces growth
^1^NVP-BEZ235 and/or ^2^SL327/U0126 [196]	GBM stem like cells	1.dual PI3K/mTOR inhibitor 2.MEK inhibitor	Akt/FOXO3a/p27 and ERK/FOXO3a/p27	TS	Prevents FOXO3a phosphorylation	In vitro	• Combination therapy caused nuclear accumulation of FOXO3a and βIII-tubulin expression as stem cell markers, inhibiting self-renewal and tumorigenicity
Hydrogen peroxide [186]	GICs	ROS	p_38_MAPK/FOXO3	TS	MAPK activates FOXO3	In vitro	• ↓Self-renewal and induces differentiation
TIC10 [197]	GBM	TRAIL inducer	TC10/TRAIL/ERK-Akt/FOXO3a/TRAIL	TS	TIC10 inactivates Akt and ERK by cooperatively inducing TRAIL	In vivo/In vitro	• ↑Survival apoptosis • Cytotoxicity against TMZ resistant cells
B10 [209]	GBM	Natural product /Botulinic acid derivative	B10/SIRT1/FOXO3a/Bim/PUMA/Bax	TS	B10 downregulates SIRT1 expression and activates FOXO3a	In vivo/In vitro	• ↑Cytotoxicity and mitochondrial dysfunction
Pt-1-DMCa [215]	GBM	platinum-maurocalcin conjugate	Src/PI3K/AKT/FOXO3a /Bim and PTEN	TS	Pt-1-DMCa induces accumulation of non-phosphorylated FOXO3a in the nucleus	In vitro	• ROS-dependent FoxO3a-mediated apoptosis • ↑PTEN
Fenofibrate [217]	GBM	lipid-lowering drug	FOXO3A/Bim	TS	FoxO3A subcellular localization	In vitro	• ↑Mitochondrial-dependent apoptosis
Icariside II [220]	GBM	Natural product and bioactive flavonoid	Akt/FOXO3a/p21 and p27	TS	ICA II inhibits the phosphorylation and activation of Akt and leads to FOXO3a nuclear translocation	In vitro	• ↓Proliferation • ↑Cell cycle arrest and apoptosis
Z-ajoene [234]	GBM stem cells	Natural product and garlic-derived compound	Akt/ FOXO3a	NA	decreased FOXO3A through dephosphorylation of AKT signaling	In vitro	• ↓ Sphere growth
Dichloroacetate (DCA) [235]	rat GSCs	An analogue of acetic acid	Foxo3	TS	DCA may increase the transcriptional activity of Foxo3	In vivo/In vitro	• ↓Proliferation • ↑ Aapoptosis

*NA* Not available, *TS* tumor supressor

## Conclusion

According to evidence collected up to now, FOXM1 acts as an absolute oncogene in gliomas, associated with poor survival, independent of the type of cell line, stage of the tumor, etc. The activity of protein kinases such as Akt, MELK, and growth factors (e.g., EGFs or FGFs) subsequently leads to phosphorylation of FOXM1 in gliomas, promoting transcriptional activity of a variety of targets, including STAT3, EZH2, β-catenin, MMP-2, Sox2, VEGF, PDGF-A, VEGF, UBE2C, Rad51, RFC5, BUB1B, Anxa1, SIRT1, ASPM, and ADAM17. Furthermore, several downregulated miRNAs, including miR-216b, miR-320, miR-370-3p, and miR-525-5p, have been verified to target 3’ UTR of FOXM1. More importantly, overexpression of FOXM1 has been strongly associated with increased proliferation, migration, angiogenesis, invasion, and resistance to radiation and TMZ in glioma through facilitating DNA repair response. Some studies have elucidated the anti-FOXM1 activity of MELK and proteasome inhibitors as well as natural products on glioma (Table 2). Therefore, it is suggested that more studies at clinical and pre-clinical levels should be conducted to assess the subsequences of FOXM1 pharmacological inhibition.

Like other FOXO subgroups, FOXO1 has a crucial role in regulating proliferation, metastasis, invasion, drug response/resistance, and apoptosis. Furthermore, while targeting the PI3K/Akt signaling pathway has a prominent role in restoring FOXO1 activity, other proteins, and transcription factors are involved in its regulation. More importantly, FOXO transcription factors exert their tumor-suppressive functions via forming a reciprocal interplay with cell cycle modulators such as CDKs and Cyclins. An overview of literature has demonstrated that FOXO1 has a controversial role in tumorgenesis of gliomas. The most oncogenic role of FOXO1 was mainly attributed to elevating the expression of stem cell markers such as OCT4 and SOX2. Altogether, focusing on the tumor suppressor role of FOXO1, most of the anticancer drugs that affect FOXO1 in glioma increase its expression, except EMAP-II and Progesterone, which their efficacy is dose or/and time-dependent. Also, the majority of these pharmacological compounds enhance FOXO1 expression through Akt, including Progesterone, Urolithin A, Xihuang Pill, and EMAP-II. Moreover, restoring FOXO1 expression could be utilized in the sensitization of tumor cells to etoposide, BCNU, or cisplatin. These findings shed light on a novel approach to conducting research and assessing FOXO1 role in the prognosis and treatment of glioma.

Finally, protein kinases such as EGFR, MAPK, IGF1R, and AKT were shown to phosphorylate FOXO3 directly or indirectly, repressing its transcriptional activity. This is while AMPK, via phosphorylating it at Ser413, causes its transactivation without affecting its subcellular localization. Moreover, MST1, via phosphorylating FOXO3, promotes its nuclear localization, leading to SIRT6 overexpression. In addition, FOXO3 was shown to act as a core component in the response of glioma cells to cellular stress, such as ROS production, hypoxia, glucose metabolism, and sirtuins. There are several upregulated ncRNAs in glioma, including miR-10b, miR-27a, miR-93, miR-155, miR-184, Circ-DONSON, and Inc-TALC that their oncogenic activity was shown to be exerted through repressing FOXO3. Therefore, suppressing their expression can be considered a step forward in restoring FOXO3 expression. Similar to FOXO1, most studies agree on the tumor-suppressive feature of FOXO3. However, under specific circumstances, both FOXO1 and FOXO3 were shown to be implicated in the occurrence of TMZ and radiation resistance.

## Future perspectives

In this review, we have covered a variety of regulatory pathways, mechanisms, and effects of FOXM1 alteration on numerous subtypes of gliomas. Most ongoing studies of FOXM1 considered it as an oncogene in light of its function in regulating several cellular processes that have been reviewed throughout this review. Moreover, among the FOX transcription factors, the FOXO subfamily is another mostly investigated one in glioma, which seems to be a tumor suppressor. Despite the vast quantity of literature describing the different mechanisms linking FOXM1 to glioma, the fact that it is a transcription factor restricts its applicability as a target compound for the therapeutic approach of glioma. Even though an enormous quantity of in vitro studies has been conducted to clarify the role of FOXM1 in glioma, the application of FOXM1 inhibition by chemical inhibitors in clinical settings has been constrained due to a number of issues, including the need for precise concentrations, and a wide range of interacting pathways and FOXM1 regulators and unknown side effects. A recent clinical trial on 79 human glioma tissues unraveled that down-regulation of FOXM1 by siRNAs induced the apoptosis, cell cycle arrest, and EMT of glioma cells [41]. Clinical trials in phases 1 and II are required to analyze the safety, pharmacodynamics, and pharmacokinetics of FOXM1 inhibitors; therefore, additional investigation and extensive clinical trials need to be conducted in order to gain conclusive evidence and elaborate the clinical potency of FOXM1 in glioma. In addition, glioma cells with mutations of the IDH gene have a decreased expression of FOMX1 compared to wild-type phenotypes. Therefore, it is suggested that the function of FOXM1 in lower-grade gliomas with IDH mutations be studied in more detail [236]. The FOXO subfamily is notably regulated by epigenetic triggers and has a close association with the cell cycle. Therefore, these proteins are exciting candidates for developing new therapeutics related to epigenetics. Further studies should investigate the function of FOXO1 and FOXO3 before and after different treatments with chemotherapy and radiotherapy in more detail. In addition, the prognostic function of these proteins should be evaluated in studies with larger sample sizes and different glioma grades, as the number of studies that evaluated the function of FOXOs in gliomas is very few and mostly based on publicly available cohorts(TCGA). The FOXO-FOXM1 axis, in particular, should be further studied in translational and clinical research due to its effects on a variety of cellular activities, including carcinogenesis, progression, and treatment resistance. Given the significance of the FOXO and FOXM1 proteins, it may be possible to utilize these proteins as potential targeted therapies and prognostic markers for glioma if their regulation mechanisms and roles in cancer initiation, progression, and drug resistance are better understood. Moreover, a combination therapy targeting the FOXO subfamily and FOXM1 has a significant chance of creating beneficial synergistic effects, reducing adverse effects, and ultimately boosting clinical outcomes. Finally, since growing numbers of models are developing in the prediction of survival of GBM patients [237, 238], the construction of models using FOX proteins for prognosis evaluation of patients appears as a promising strategy in clinical settings, and future studies should consider this point.

## Data Availability

Data sharing does not apply to this article as no datasets were generated or analyzed during the current study.
